# The oxoglutarate dehydrogenase complex is involved in myofibril growth and Z-disc assembly in *Drosophila*

**DOI:** 10.1242/jcs.260717

**Published:** 2023-06-30

**Authors:** Nicanor González Morales, Océane Marescal, Szilárd Szikora, Anja Katzemich, Tuana Correia-Mesquita, Péter Bíró, Miklos Erdelyi, József Mihály, Frieder Schöck

**Affiliations:** ^1^ Department of Biology, McGill University, Quebec H3A 1B1, Canada; ^2^ Department of Biology, Dalhousie University, Nova Scotia B3H 4R2, Canada; ^3^ Institute of Genetics, Biological Research Centre, Hungarian Academy of Sciences, Szeged 6726, Hungary; ^4^ Department of Optics and Quantum Electronics, University of Szeged, Szeged 6720, Hungary; ^5^ Department of Genetics, University of Szeged, Szeged 6726, Hungary

**Keywords:** *Drosophila*, Muscle, Myofibril, Ogdh, TCA cycle, Zasp

## Abstract

Myofibrils are long intracellular cables specific to muscles, composed mainly of actin and myosin filaments. The actin and myosin filaments are organized into repeated units called sarcomeres, which form the myofibrils. Muscle contraction is achieved by the simultaneous shortening of sarcomeres, which requires all sarcomeres to be the same size. Muscles have a variety of ways to ensure sarcomere homogeneity. We have previously shown that the controlled oligomerization of Zasp proteins sets the diameter of the myofibril. Here, we looked for Zasp-binding proteins at the Z-disc to identify additional proteins coordinating myofibril growth and assembly. We found that the E1 subunit of the oxoglutarate dehydrogenase complex localizes to both the Z-disc and the mitochondria, and is recruited to the Z-disc by Zasp52. The three subunits of the oxoglutarate dehydrogenase complex are required for myofibril formation. Using super-resolution microscopy, we revealed the overall organization of the complex at the Z-disc. Metabolomics identified an amino acid imbalance affecting protein synthesis as a possible cause of myofibril defects, which is supported by OGDH-dependent localization of ribosomes at the Z-disc.

## Teaser

**Summary:** Myofibrils in muscles are very regular structures. We show that the scaffolding protein Zasp52 recruits the oxoglutarate dehydrogenase complex to coordinate myofibril growth and maintain their regularity.

## INTRODUCTION

Striated muscles are long contractile cables that bridge two rigid structures, such as bones or regions of the exoskeleton. Muscle contraction is responsible for providing the energy required to displace those rigid units and thus animal movement. Muscles are formed by long intracellular cables, called myofibrils, that bridge the ends of muscles; the coordinated shortening of these myofibrils causes muscle contraction (Ahmed et al., 2022). Myofibrils are themselves composed of tandemly repeated units called sarcomeres. Sarcomeres are built up from a complex array of antiparallel actin and myosin filaments, where actin filaments are anchored to the flanks of the sarcomere at a protein complex called the Z-disc, whereas the myosin filaments are anchored at the center of the sarcomere at another protein complex called the M-line. Coordinated shortening of the sarcomeres produces myofibril contraction (Ahmed et al., 2022; Dos Remedios and Gilmour, 2017; Gunage et al., 2017; Lemke and Schnorrer, 2017; Luis and Schnorrer, 2021; Nikonova et al., 2020).

Because all sarcomeres contract in synchrony, their sizes are identical, and muscles use a variety of ways to ensure sarcomere size homogeneity. This is particularly true for the indirect flight muscle (IFM), a special muscle that evolved in insect lineages to sustain high-frequency contractions for prolonged periods. One of the adaptations of the IFM is very regular sarcomeres with a very small contractile range. In *Drosophila*, the IFM develops during the early pupal stages and then rapidly grows to fill most of the thoracic space during the late pupal stages (Reedy and Beall, 1993). Muscle growth is a very coordinated process. Myofibrils first form very thin longitudinal cables that stably grow by recruiting cytoplasmic proteins (Katzemich et al., 2013; Loison et al., 2018; Reedy and Beall, 1993; Spletter et al., 2018). The M-line and Z-disc grow together with the myofibril, actively mediating myofibril growth (González-Morales et al., 2019b; Katzemich et al., 2012, 2013; Orfanos et al., 2015).

Local and global mechanisms control muscle growth and sarcomere homogeneity. Local mechanisms acting on individual myofibrils or sarcomeres control sarcomere length, myofibril width and length of the I-band (the region that contains Z-discs and is devoid of myosin filaments). Sarcomere length is controlled by the length of the connecting protein titin (Tskhovrebova and Trinick, 2017) and the fine regulation of actin and myosin dynamics (Molnar et al., 2014; Shwartz et al., 2016). The length of the I-bands is controlled by the function of the Lasp proteins (Fernandes and Schöck, 2014). Myofibril width is set in place by controlled oligomerization of Zasp proteins at the Z-disc (González-Morales et al., 2019b). In contrast to these, global mechanisms act on the whole muscle, containing thousands of sarcomere units. One example of a global mechanism is the muscle growth coordination imposed by the continuous increase in tissue tension. As muscles grow, tension builds because of premature muscle contractions, and the increase in tendon and cuticle stiffness (Chu and Hayashi, 2021; Lemke and Schnorrer, 2017; Weitkunat et al., 2017, 2014). This continuous increase in tension coordinates the growth and the shape of myofibrils and mitochondria (Avellaneda et al., 2021). Other global mechanisms include growth regulation by the Hippo pathway (Kaya-Copur et al., 2021), the role of the E2F–DP heterodimeric transcription factor (Zappia and Frolov, 2016; Zappia et al., 2019) and the role of insulin (Demontis and Perrimon, 2009). The Hippo pathway and E2F–DP separately coordinate myofibril and mitochondrial growth rates by promoting the expression of myofibril and mitochondria proteins (Kaya-Copur et al., 2021; Zappia et al., 2019). Despite all these well-described mechanisms, the interconnections between the global growth cues and the local growth mechanisms have yet to be deciphered.

To gain insights into the mechanisms of Zasp proteins promoting myofibril growth, we screened for Z-disc proteins recruited by Zasp involved in myofibril diameter size regulation. Zasp proteins are members of the ALP/Enigma family; they consist of a PDZ, a ZM and up to 4 C-terminal LIM domains. They localize to the Z-disc, with their LIM domains at the very center of the disc and their PDZ domain at the periphery (Katzemich et al., 2013; Szikora et al., 2020). Through the PDZ domain, Zasp binds actinin and establishes the structural core of the Z-disc (Liao et al., 2016). Actinin anchors actin filaments from opposing sarcomeres. Zasp exists in two forms, a blocking and a growing form, with opposite roles during myofibril diameter growth (González-Morales et al., 2019a,b; Katzemich et al., 2013). The blocking forms prevent the recruitment of the growing isoforms to the Z-disc, whereas the growing isoforms recruit Zasp proteins to the Z-disc. The self-association of Zasp is mediated by a physical interaction between its LIM domains and ZM domain (González-Morales et al., 2019b).

We report that the E1 subunit of 2-Oxoglutarate Dehydrogenase (OGDH/E1), a crucial enzyme in the tricarboxylic acid (TCA) cycle, is recognized by the LIM domains of Zasp. OGDH/E1 is recruited to the growing Z-disc in addition to its mitochondrial localization, and its function is required for myofibril growth and assembly. The TCA cycle is a loop of chemical reactions and constitutes a metabolic buffering system. Anaplerotic metabolic pathways replenish the TCA cycle, whereas cataplerotic reactions use the TCA cycle metabolites (Martinez-Reyes and Chandel, 2020; Owen et al., 2002). An important step in the TCA cycle is the conversion of 2-oxoglutarate into succinyl-CoA, which is catalyzed by the OGDH complex – a giant enzyme cluster composed of multiples of three subunits (Tretter and Adam-Vizi, 2005). The dihydrolipoyllysine-residue succinyltransferase subunit (DLST, also known as E2 or CG5214) serves as a structural core unit (Skalidis et al., 2020). The OGDH (also known as E1 or Nc73EF) and dihydrolipoyl dehydrogenase (DLD, also known as E3 or CG7430) subunits sit in the periphery of the E2 core (Larkin et al., 2021; Skalidis et al., 2020; Tretter and Adam-Vizi, 2005) (Chen et al., 2015; Gruntenko et al., 1998; Yoon et al., 2017). The OGDH (also known as E1) subunit recognizes the 2-oxoglutarate substrate and provides specificity to the complex (Tretter and Adam-Vizi, 2005). This enzyme complex, therefore, fulfills crucial functions in mitochondria, providing metabolites and reduced electron carriers for oxidative phosphorylation. Here, we propose an additional role for the OGDH complex in providing amino acids for protein synthesis to sustain myofibril growth and Z-disc assembly. Together, these data suggest a novel link between a local Zasp-dependent myofibril growth control mechanism and a global myofibril growth control mechanism: amino acid availability.

## RESULTS

**Fig. 1.**
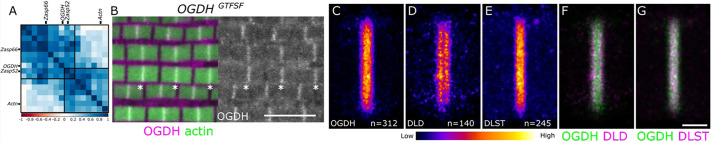
**OGDH localizes to the Z-disc.** (A) Heatmap showing the ERC values from all the hits in the ERC screen ordered by automatic clustering. Note the Zasp52/Zasp66 and the Zasp52/actinin clusters. The color scale of the ERC values from −1 to +1 is at the bottom. The positions of Actn, Zasp52, Zasp66 and OGDH are indicated. (B) Confocal image of muscles carrying the *OGDH^MI06026-GFSTF.1^* GFP trap allele. OGDH-GFP (magenta) and actin filaments (green); OGDH-GFP localizes to the Z-disc. Composite and single-channel images are shown. Asterisks show selected Z-discs in B. Scale bar: 5 µm. (C-G) Super-resolution dSTORM images of the individual subunits of the OGDH complex as intensity heat maps (see also Fig. S2C). (C) The E1 subunit, OGDH, localizes to a wide band at the Z-disc core. (D) The DLD subunit localizes to two discrete bands that overlap the periphery of the E1 subunit distribution. (E) The E2 core subunit DLST localizes to a wide band at the Z-disc core in the same way as the E1 subunit does. (F,G) Composite dSTORM images: OGDH with DLD (F) and OGDH with DLST (G). Scale bar: 500 nm. The GFP-tagged versions of the subunits were used together with a GFP antibody.

Because Zasp proteins are often insoluble, we used a bioinformatic approach to look for proteins with similar evolutionary rates to the Zasp proteins in order to find proteins recruited by Zasp to the growing Z-disc. Evolutionary rate covariation (ERC) is a measurement of shared evolutionary history between proteins (Clark and Aquadro, 2010; Raza et al., 2019). We retrieved the ERC values for Zasp52, Zasp66 and Actn (α-actinin), and matched them to all *Drosophila* proteins with available ERC values (roughly 11,100 proteins) from a previously characterized project (Findlay et al., 2014). We found 16 proteins with ERC values greater than 0.5, with at least two of the three bait proteins. We then used an automatic clustering method to group the candidate proteins. Two clusters were obtained, the Zasp52/Zasp66 and the Zasp52/actinin groups (Fig. 1A), possibly reflecting the two roles of Zasp52: stabilization of actinin and recruitment of other Zasp proteins. As we primarily aimed for Z-disc proteins, we investigated which of the candidate proteins colocalizes with Zasp and actinin at the Z-disc. To do so, we obtained tagged versions of the candidate proteins and analyzed their intracellular localization. To avoid the signal coming from the mitochondria, we used a glycerol washing step to remove the mitochondria (Xiao et al., 2017). From the candidates, OGDH (also known as Nc73EF) had the most obvious Z-disc localization compared with the negative control (Fig. 1B). We then confirmed the presence of OGDH at the Z-disc by co-immunoprecipitation (IP). Because Zasp52 is highly insoluble in traditional immunoprecipitation (IP) buffers, we used the well-characterized Zasp-binding protein α-actinin. OGDH-GFP purified from adult thoraces precipitates with α-actinin, confirming the presence of OGDH at the Z-disc (Fig. S1). Oxoglutarate dehydrogenase (OGDH) is a crucial enzyme in the TCA cycle (Martinez-Reyes and Chandel, 2020).

Because OGDH localization at the Z-disc was unexpected, we decided to further study its role in myofibril assembly. First, we tested whether the GFP tag faithfully reflects OGDH localization by using a smaller FLAG tag that is less likely to cause localization artifacts. Using the UAS/Gal4 system, we expressed OGDH-FLAG and human OGDH-FLAG in the IFM, and analyzed their localization in glycerinated muscles using immunohistochemistry. Both forms localize mainly to the Z-disc, but also slightly to the M-line, suggesting that the Z-disc localization we observed in OGDH-GFP flies is not an artifact caused by the GFP (Fig. S1).

OGDH is the E1 subunit of the OGDH complex, the other two subunits are DLD and DLST. We therefore tested the localization at the Z-disc of all three subunits. We used GFP-tagged genomic versions of OGDH, DLD and DLST, and to determine the precise localization of the subunits within the Z-disc, we used a previously validated dSTORM (direct stochastic optical reconstruction microscopy) super-resolution microscopy method (Szikora et al., 2020). Overall, we found that all the subunits are present at the Z-disc. The small differences in distribution patterns provide clues about the structure of the complex at the Z-disc. (Fig. 1C-E). The distribution of DLST, the core subunit, and the OGDH molecules are largely overlapping (Fig. 1C,E,G). The third subunit, DLD, also accumulates at the Z-disc, but it localizes into two discrete bands alongside the Z-disc (Fig. 1D). The double-band pattern of DLD still exhibits a significant overlap with that of OGDH (Fig. 1F); therefore, these super-resolution data suggest that two OGDH complexes assemble at either side of the Z-disc center, with the DLD subunits concentrated on the outer periphery (Fig. S2).

**Fig. 2.**
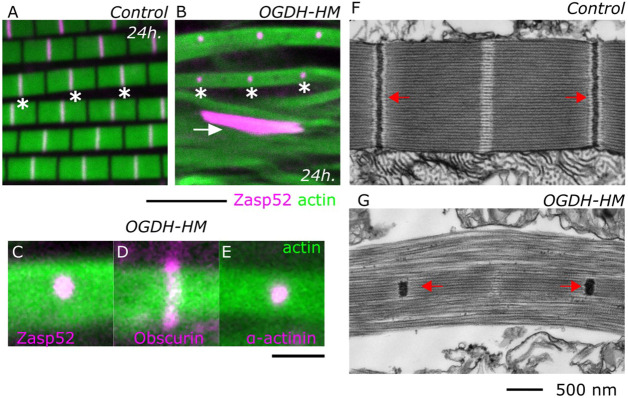
**Depletion of OGDH results in a small Z-disc.** (A,B) Confocal images of control (A) and OGDH-HM (B) muscles with Zasp52-mCherry as a Z-disc marker (magenta) and actin filaments as a reference (green). In OGDH-HM muscles, the Z-discs do not grow to their final size and appear small. Protein aggregates are occasionally present (arrow). (C-E) High-magnification confocal images of OGDH-HM muscles with different sarcomere markers. In C, the Z-disc marked by Zasp52-mCherry is smaller than the myofibril. In D, the M-line marked by Obscurin-GFP covers the entire width of the myofibril. In E, the Z-disc marked by α-actinin-GFP is smaller then the myofibril. In D and E, actin filaments are shown in green. (F,G) Transmission electron microscopy images of control and OGDH-HM sarcomeres. The Z-discs are the black electron-dense structures indicated by red arrows. The H zone is the white region at the center of the sarcomere. In the control image, the Z-disc is well defined and spans the whole diameter of the sarcomere. In OGDH-HM muscles, the Z-disc is reduced to a tiny clump at the middle of the myofibril (arrows). Asterisks indicate selected Z-discs in A and B. Scale bars: 5 µm in A and B; 500 nm in C and D. Flies are less than 2 days old.

We then analyzed the consequences of inactivating OGDH. We removed OGDH from the developing indirect flight muscles using Act88f-Gal4 in combination with an RNAi directed against all *OGDH* isoforms (HMS00554; we refer to these flies as OGDH-HM). We noted that all the OGDH-HM flies were flightless, and all their myofibrils were abnormal when compared with controls (Fig. 2A,B). OGDH-HM muscles had very small Z-discs and occasionally protein aggregates were visible in the cytoplasm (Fig. 2B, asterisk and arrow). These protein aggregates are Z-disc aggregates, as shown by the presence of Zasp66 and actinin, and the absence of the M-line marker obscurin (Fig. S3). Importantly, the OGDH defects are specific to the Z-disc, because the M-line marker obscurin crosses the entire myofibril (Fig. 2D). In addition, we used transmission electron microscopy to characterize the phenotype in more detail, and we noted that the Z-discs in OGDH-HM flies, which are located at the center of the fibrils, were severely reduced in size (Fig. 2F,G), suggesting a Z-disc growth or assembly defect. This results in unattached thin filaments that invade the H zone; however, the overall width of myofibrils is largely maintained, presumably because obscurin still tethers myosin filaments at the M-line (Fig. 2D).

To test the specificity of the RNAi knockdown, we used other approaches to reduce the function of OGDH in the muscles. We used two other RNAi lines that target different sequences of the *OGDH* gene (GD12778 and GD50393), and an indirect flight muscle-specific CRISPR-Cas9-based method targeting the *OGDH* gene (OGDH-CRISPR, TKO.GS00550). All these conditions had muscles with smaller Z-discs and protein aggregates (Fig. S3), confirming the OGDH requirement for proper myofibril and Z-disc growth. The Z-discs were smaller in the case of OGDH-HM and OGDH-CRISPR than in the two other RNAi conditions, whereas the aggregates were most common in OGDH-CRISP, followed by OGDH-HM, GD50393 and GD12778 (Fig. S3). Thus, overall, all four methods resulted in similar muscle defects, and the slight differences observed likely reflect the effectiveness of reducing OGDH levels in different conditions.

**Fig. 3.**
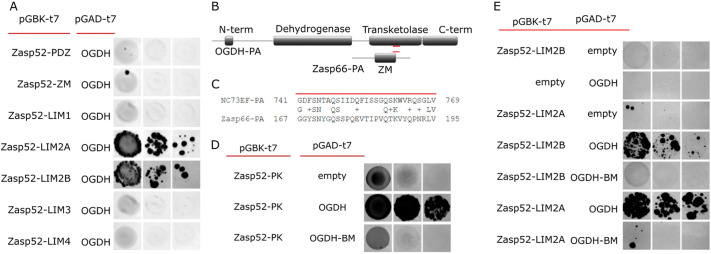
**The LIM2 domain of Zasp52 binds OGDH through a ZM-like sequence.** (A) Y2H assays show LIM2a and LIM2b are the only domains in Zasp52 that bind OGDH. (B) Cartoon of OGDH-PA and Zasp66-PA proteins with protein domains highlighted. The short sequence common to both proteins is marked by a red line. OGDH-BM has this short sequence removed. (C) Alignment of the common sequence between OGDH-PA and Zasp66-PA isoforms. (D) Y2H assays show that Zasp52-PK binds to OGDH but the interaction is lost in OGDH-BM, a mutant version that lacks the common short sequence. (E) Y2H assays show LIM2a and LIM2b bind the short sequence in OGDH. Serial dilutions are shown from left to right in A, D and E (OD 0.1, OD 0.01 and OD 0.001).

After the initial phenotypic characterization of OGDH, we explored the physical association between Zasp52 and OGDH proteins. First, we used a yeast two-hybrid assay to test for protein-protein binding. Yeast expressing Zasp52 and OGDH grow on selective media, suggesting a physical interaction, whereas yeast expressing Zasp66 or actinin together with OGDH did not grow (Fig. 3 and data not shown). We then used this assay to find the binding site mediating the interaction between OGDH and Zasp52. First, we paired OGDH with all the possible individual domains of Zasp52. Only Zasp52-LIM2a and -LIM2b together with OGDH restored yeast growth (Fig. 3A). We have previously shown that the LIM domains of Zasp52 bind to the ZM domains of Zasp proteins (González-Morales et al., 2019b). We noted a region in OGDH that weakly aligns with the ZM domain of Zasp66 and asked whether this sequence would also mediate OGDH-Zasp52 binding (Fig. 3B,C). We made a mutant OGDH version (referred to as OGDH-BM, short for binding mutant) that lacks this sequence, and we found that Zasp52 was unable to bind OGDH-BM (Fig. 3D). The LIM2a or LIM2b domains are also unable to bind OGDH-BM in Y2H assays (Fig. 3E).

**Fig. 4.**
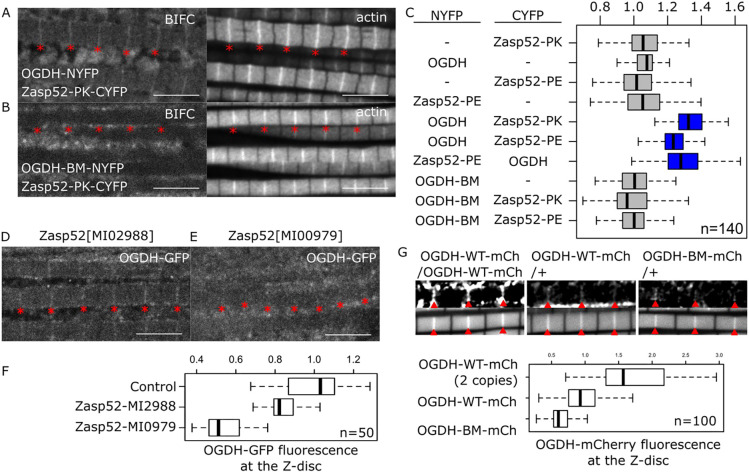
**Zasp52 recruits OGDH to the Z-disc.** (A,B) Confocal images of muscles showing BiFC fluorescence and actin staining for reference. (A) Zasp52-PK and OGDH form a complex at the Z-disc; BiFC signal is detected at the Z-disc (asterisks). (B) OGDH-BM does not bind Zasp52-PK at the Z-disc, and the BiFC signal is not detected at the Z-disc. (C) Plots of the BiFC fluorescence intensity values relative to background noise. Physical interaction is detected between Zasp52 and OGDH (blue) (*P*<0.0001 in all cases). (D,E) Confocal images showing the localization of OGDH-GFP in two *Zasp52* mutants. (D) In *Zasp52^MI02988^* mutants, OGDH is properly localized to the Z-disc. (E) In *Zasp52^MI00979^* mutants, OGDH-GFP is not detected at the Z-disc (asterisks). (F) Boxplot of OGDH-GFP intensities in control and *Zasp52* mutant backgrounds. The *Zasp52^MI00979^* mutant has reduced Z-disc localization compared with the wild type and *Zasp52^MI02988^* mutant (*P*=2e-29 and *P*=2e-27). (G) Confocal images of wild-type or binding mutant OGDH forms tagged with mCherry and box plot of the fluorescence intensity. Flies with one or two copies of OGDH-wt-mCh show Z-disc localization. The OGDH-BM-mCh form has reduced Z-disc localization compared with the wild type (*P*=4e-11). Red arrowheads indicate the Z-discs. Scale bars: 5 µm. *P*-values were calculated using Welch's two-sample *t*-test followed by a Bonferroni correction. For box plots, the box represents the 25–75th percentiles, and the median is indicated. The whiskers show the minimum and maximum values.

Because both forms localize to the Z-disc when overexpressed, we used bimolecular fluorescence complementation assays to test the direct interaction between Zasp and OGDH at the Z-disc (Marescal et al., 2020). Muscles expressing OGDH fused to the N-terminal region of YFP, and Zasp52 fused to the complementary C-terminal region of YFP showed a clear fluorescent signal at the Z-disc (Fig. 4A and Fig. S4), suggesting OGDH and Zasp bind at the Z-disc. Importantly, the signal is lost in OGDH-BM conditions (Fig. 4B,C and Fig. S4). We then tested the role of Zasp52 in recruiting OGDH to the Z-disc. We imaged OGDH-GFP in two *Zasp52* mutants. *Zasp52^MI02988^* affects the PDZ domain but not the LIM domains, and *Zasp52^MI00979^* forces a stop before the last three LIM domains but leaves the PDZ domain intact. OGDH localizes to the Z-disc in control and *Zasp52^MI02988^* mutant muscles but not in *Zasp52^MI00979^* mutants (Fig. 4D-F), further supporting the involvement of the LIM domains in OGDH binding.

Because overexpression studies are prone to subcellular localization artifacts, we also analyzed the endogenous OGDH protein. To this end, we created OGDH mutant alleles by incorporating a mCherry tag either into the wild-type OGDH or the OGDH-BM form (*OGDH-WT-mCh* and *OGDH-BM-mCh*, respectively). First, we tested the correct mitochondrial localization of both OGDH forms in the IFM by avoiding the glycerinated step during sample preparation. As expected, both *OGDH-WT-mCh* and *OGDH-BM-mCh* strongly localize to the mitochondria (Fig. S5). Homozygous *OGDH-WT-mCh* flies are viable and their myofibrils develop properly. In contrast, homozygous *OGDH-BM-mCh* is lethal. As heterozygotes, both alleles develop normal myofibrils. Because the mitochondrial signal is strong, it masks the Z-disc signal, so we used glycerinated muscles, to test the Z-disc localization of OGDH-BM-mCh. The Z-disc localization of OGDH-BM-mCh is diminished roughly by half compared with the OGDH-WT-mCh control (Fig. 4G), indicating that Zasp binding is at least partially required for Z-disc localization.

**Fig. 5.**
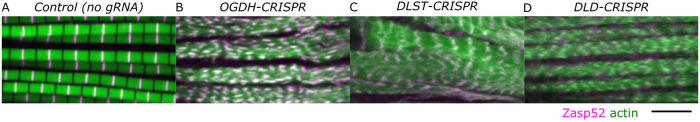
**Myofibril defects observed after targeting the individual OGDH complex subunits through tissue-specific CRISPR-Cas9.** (A-D) Confocal images of muscles with CRISPR-induced mutations of the OGDH complex subunits. Flies are 1-2 days old. Negative control without a gRNA (A), the E1 subunit OGDH (B), the E2 subunit DLST (C) and the E3 subunit DLD (D). Mutations in any of the OGDH complex subunits result in very thin myofibrils that tend to stack on each other. Zasp52 marks the Z-disc in magenta and actin filaments are shown in green. Scale bar: 5 µm.

Because OGDH normally functions as one of the three subunits of the OGDH protein complex, we used tissue-specific CRISPR-Cas9 targeted mutagenesis to examine the function of the three OGDH complex subunits. We expressed Cas9 together with gRNAs targeting *OGDH*, *DLST* or *DLD* in the indirect flight muscles and observed a very similar sarcomere phenotype in all three cases, characterized by a strong impairment of myofibrillar organization and sarcomere structure (Fig. 5). Collectively, these data strongly argue that the OGDH complex is crucial for proper sarcomere arrangement, including Z-disc organization.

**Fig. 6.**
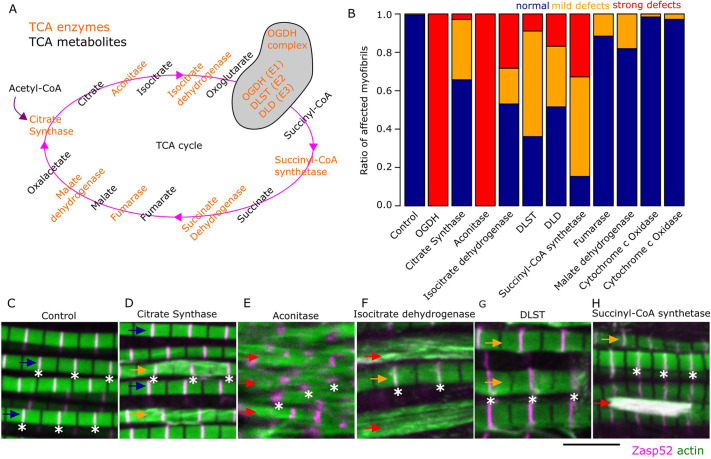
**Myofibril defects observed after depletion of individual TCA cycle enzymes in the IFM.** (A) Cartoon of the TCA cycle in *Drosophila*; the enzyme-encoding genes are orange and the metabolites are black. The TCA cycle is a loop of chemical reactions and constitutes a metabolic buffering system. The cycle starts when citrate synthase combines acetyl-CoA with oxaloacetate. Aconitase then converts citrate into isocitrate, followed by two oxidative decarboxylation steps. The homodimeric isocitrate dehydrogenase converts isocitrate into oxoglutarate. In the second decarboxylation step, the OGDH complex, which is composed of three subunits (OGDH/E1, DLST/E2 and DLD/E3), converts oxoglutarate into succinyl-CoA, which is converted to succinate by succinyl-CoA synthetase. Succinate dehydrogenase, which is also part of the electron transport chain, then oxidizes succinate to fumarate. Fumarase converts fumarate to malate and, finally, malate dehydrogenase reconstitutes the oxaloacetate. (B) Stacked bar plot of the ratio of myofibrils that appear normal (blue), have mild defects (orange) and have strong defects (red) in different RNAi conditions. (C-H) Confocal images showing the myofibril phenotypes of select TCA cycle enzymes. Flies are 1-2 days old. Examples of myofibril defects are indicated by colored arrows that match the colors used in B. Asterisks indicate the position of selected Z-discs. (C) The control genotype (*Act88F-Gal4, Zasp52-mCherry*). (D) Depletion of citrate synthase causes myofibril streaming (orange arrows). (E) Depletion of aconitase causes a complete loss of myofibril structure (red arrows). (F) Depletion of isocitrate dehydrogenase causes loss of myofibril structure (red arrows). (G) Depletion of the DLST (E2) subunit causes strong myofibril defects (orange arrows). (H) Depletion of succinyl-CoA synthetase causes a loss of myofibril structure (red arrow) and myofibril defects (orange arrow). In C-H, Zasp52 marks the Z-discs in magenta. Actin filaments are shown in green. Scale bar: 5 µm.

Given the crucial role of OGDH in the TCA cycle, we next asked whether the depletion of other TCA cycle components would affect sarcomere assembly. We expressed RNAi transgenes targeting the major components of the TCA cycle (Fig. 6A) and, strikingly, we observed sarcomere phenotypes in 83% of them (Fig. 6B-H, Table S1). Most notably, Aconitase, Isocitrate Dehydrogenase, OGDH complex E2 subunit and Succinyl CoA-Synthetase, which catalyze sequential steps in the cycle, had the most dramatic effects (Fig. 6B), often exhibiting a complete disintegration of the myofibril structure (Fig. 6E,F,H, red arrows) or myofibrils with more subtle defects (Fig. 6D,F-H, orange arrows). In the case of the few TCA components that do not affect sarcomere structure, enzyme redundancy is a likely explanation for the lack of phenotype. As the TCA cycle fuels the electron transport chain, which generates ATP, we also tested the role of ATP by analyzing muscles with a compromised electron transport chain. Removing Cox5a, an essential subunit of the Cytochrome c Oxidase (Mandal et al., 2005), with two different RNAi constructs, does not result in myofibril defects, suggesting that the electron transport chain does not account for the defects observed in the TCA cycle enzymes (Fig. 6B). We asked whether other TCA cycle enzymes localize to the Z-disc. We focused on Aconitase and Isocitrate Dehydrogenase because they are the two enzymes that directly precede OGDH and tagged versions are available. We used a GFP version of Aconitase (*Acon1^CC00758^*) and a Flag-tagged version of Isocitrate Dehydrogenase (UAS-Idh-FLAG) to test their localization. Acon1 has a faint Z-disc fluorescence signal, while Idh has a strong Z-disc signal, suggesting that both are present at the Z-disc (Fig. S6).

**Fig. 7.**
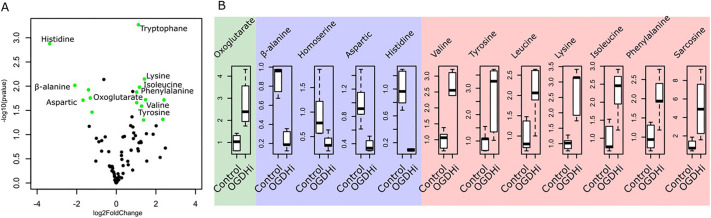
**Gas chromatography-mass spectrometry metabolomic analysis of OGDH-HM reveals a defect in amino acid balance.** (A) Volcano plot of all the metabolites analyzed in control (Act88F-Gal4, Zasp52-GFP) and OGDH-HM (Act88F-Gal4, UAS-OGDH-HM, Zasp52-GFP) samples. Significance −log10(*P*-value) and fold-change log2 are on the *y*- and *x*-axis, respectively. Each dot is a unique metabolite; the green dots are metabolites with *P*<0.05 and absolute log2FoldChange values>1. The names of selected metabolites are shown. There is an accumulation of oxoglutarate and an imbalance in many amino acids. (B) Box plots of selected metabolites in control and OGDH-HM (OGDHi) samples. The green panel shows oxoglutarate is approximately three times more concentrated in OGDH-HM than in controls, validating the approach. The blue panel shows the reduction of β-alanine, homoserine, aspartic acid and histidine in OGDH-HM compared with the control samples. The red panel shows the accumulation of several amino acids. Sarcosine is an amino acid intermediate of glycine metabolism. The box represents the 25–75th percentiles, and the median is indicated. The whiskers show the minimum and maximum values.

The TCA cycle is a metabolic hub that connects many aspects of cellular homeostasis including the biosynthesis of many amino acids (Martinez-Reyes and Chandel, 2020). To investigate how the loss of OGDH affects muscle metabolism, we performed a metabolomic analysis of OGDH-HM and control muscles (Fig. 7A). Consistent with a role in the TCA cycle, we observed a 2.7-fold increase in oxoglutarate accumulation, indicating the sensitivity of the approach. Interestingly, this change was paralleled with abnormal levels of numerous amino acids. The most extreme cases are Histidine and β-alanine, which are almost absent compared to control muscles (Fig. 7A and B). Homoserine and aspartic acid are also less abundant in OGDH-HM muscles (Fig. 7B), whereas valine, tyrosine, leucine, lysine, isoleucine, phenylalanine and sarcosine, a glycine intermediate, accumulate in OGDH-HM muscles (Fig. 7B). Overall, these data suggest a widespread effect on amino acid metabolism.

**Fig. 8.**
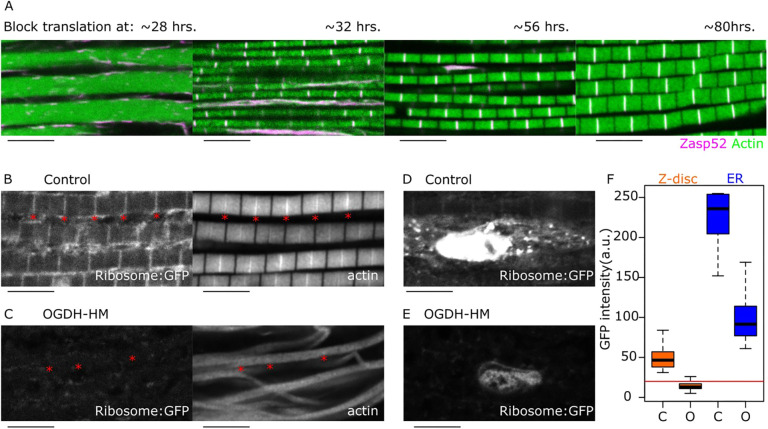
**A general block in protein synthesis results in smaller but otherwise normal myofibrils.** (A) IFM in which general translation is inhibited during metamorphosis for a maximum time of 48 h. The time at which the translation block was initiated is indicated at the top of each image. Blocking ribosome function using temperature-sensitive ricin at different time points results in the differential blocking of myofibril diameter growth. Blocking ribosome function at ∼28 h after pupa formation (APF) completely abolishes striation. Blocking ribosome function at ∼32 h APF results in myofibril striation, but diameter growth is impaired. Blocking ribosome function at ∼56 h APF results in smaller diameters but otherwise normal myofibrils. Blocking ribosome function at ∼80 h APF does not affect myofibril appearance much. (B-F) Confocal images of control and OGDH-HM muscles expressing a GFP-tagged version of RpS5a (Ribosome-GFP); actin filaments are also shown. Flies are 1-2 days old. (B) In control samples (Act88F-Gal4, UAS-RpS5a-GFP), ribosomes are spread in the cytoplasm but accumulate at the Z-disc. (C) In OGDH-HM samples (Act88F-Gal4, UAS-RpS5a-GFP, UAS-OGDH-HM), ribosomes are mostly absent. Red asterisks in B and C indicate selected Z-discs. (D) In controls, ribosome-GFP is also detected in the region surrounding the nucleus. (E) In OGDH-HM, ribosome-GFP is present in the ER. Scale bars: 5 µm. (F) Box plot of ribosome-GFP intensities in Z-disc and ER in control (C) and OGDH-HM (O) muscles. GFP intensity in OGDH-HM muscles is reduced at the Z-disc and ER (*P*<2.2e-16 using Welch's two-sample *t*-test). The red line indicates the noise level. The box represents the 25–75th percentiles, and the median is indicated. The whiskers show the minimum and maximum values.

We then hypothesized that problems in protein synthesis could cause the myofibril and Z-disc defects in OGDH-depleted muscles. It is well known that when amino acids are missing, cells actively block protein synthesis by reducing ribosome biogenesis or by actively degrading them (Destefanis et al., 2020; Iadevaia et al., 2014). To test this possibility, we artificially blocked global protein synthesis by expressing the A subunit of the ricin toxin in muscles during different developmental periods (Moffat et al., 1992). Ricin expression during early development results in a complete absence of sarcomere structure (Fig. 8A, ∼28 h). In contrast, ricin expression slightly after the first appearance of sarcomeres and before sarcomere growth blocks sarcomere growth (Fig. 8A, ∼32 h and ∼56 h). Importantly, this results in normal myofibrils that are only reduced in width, not in the Z-disc-specific defects seen in OGDH mutants. Finally, ricin expression after the growth period of ∼80 h has little effect on sarcomere structure or size (Fig. 8A, ∼80 h). We then asked whether the number of ribosomes was affected by OGDH depletion. We used a GFP-tagged version of the ribosomal protein RpS5a (Kong et al., 2019). In control muscles, RpS5a localized to the perinuclear region and the Z-discs (Fig. 8B,D); however, in OGDH-depleted muscles, RpS5a fluorescence was strongly reduced, especially at the Z-disc (Fig. 8C,E,F). This suggests partial ribosome degradation or a termination in ribosome biogenesis.

## DISCUSSION

Through a bioinformatic and Z-disc localization screen for myofibril growth regulators, we found the E1 subunit of the OGDH complex. We found that OGDH-E1 is a Z-disc and mitochondrial component required for the growth and assembly of myofibrils. Through a metabolomic analysis, we found that OGDH is required for the synthesis of some amino acids that we propose are required for myofibril growth.

### Unexpected localization of the OGDH complex at the Z-disc

Although metabolic enzymes are not typically recognized as myofibril components, their presence at the Z-disc is not entirely unprecedented. Six glycolytic enzymes that catalyze consecutive reactions along the glycolytic pathway localize to the Z-disc (Sullivan et al., 2003; Wojtas et al., 1997). Our data, together with previous data on glycolytic enzymes, suggest that the Z-disc may be a common space for metabolic reactions that take place in the cytoplasm of muscles. Similarly, an interactome study using titin-BioID knock-in mice showed the presence of several enzymes at the Z-disc (Rudolph et al., 2020). Overall, our work demonstrates that the OGDH complex and at least two other TCA cycle enzymes localize to the Z-disc.

Using dSTORM super-resolution imaging, we show that the three subunits of the OGDH complex localize at the Z-disc and assemble into an asymmetric configuration in which the E3 subunit lies at the periphery of the complex. This is consistent with asymmetries observed by cryo-EM models of native α-keto acid dehydrogenase complexes from the *Chaetomium thermophilum* fungus (Kyrilis et al., 2021). Our work shows for the first time the asymmetry of the OGDH complex in animals.

### OGDH is a novel Zasp-binding protein

Zasp proteins exist in two isoforms: the ones that contain LIM domains were designated as growing isoforms, while the ones that do not are called blocking isoforms (Katzemich et al., 2013; Liao et al., 2016). The growing isoforms recruit both growing and blocking forms through their LIM domains. The LIM domains bind the ZM domain present in both forms (González-Morales et al., 2019b). Here, we show that the LIM2 domain of Zasp52 recognizes the OGDH protein through a sequence very similar to the ZM domain. Interestingly, although all LIM domains can bind Zasp66, only the LIM2 domain binds OGDH, indicating that the other LIM domains might be used exclusively for Zasp proteins or for yet unidentified Z-disc proteins. Screening for proteins with ZM-like regions might be a strategy to find other Zasp-binding proteins. OGDH-BM-mCherry, which lacks the Zasp52 binding site, shows reduced localization at the Z-disc.

### Control of myofibril growth

As sarcomere homogeneity is crucial for muscle function, several pathways coordinate *Drosophila* IFM growth. At the global muscle level, the Hippo pathway controls the rapid growth burst of the flight muscles by controlling the expression of many of the major sarcomere proteins (Kaya-Copur et al., 2021). The activating function of RBf with the E2F–DP heterodimeric transcription factor promotes the postmitotic expression of sarcomere proteins required for myofibril growth (Zappia and Frolov, 2016; Zappia et al., 2019), and the Insulin and mTOR signaling pathways regulate endoreplication, which is required for postmitotic growth (Demontis and Perrimon, 2009). At the local myofibril level, the Zasp oligomerization pathway then controls myofibril diameter (González-Morales et al., 2019b), and a combination of titin filaments and actin regulators set the sarcomere length (Molnar et al., 2014; Shwartz et al., 2016; Tskhovrebova and Trinick, 2017). Finally, some myofibril proteins are translated locally at the Z-disc (Denes et al., 2021). Here, we propose a link between the Zasp oligomerization system and the OGDH complex, which lies at the core of the TCA cycle. We propose a feedback system between Zasp oligomerization and the local amino acid pool that provides robustness to the myofibril diameter size control.

### Mitochondrial versus Z-disc functions

The majority of OGDH and other TCA cycle enzymes localize to mitochondria where they are involved in two main functions: generating reduced electron carriers for eventual ATP production; and providing metabolic intermediates, mostly amino acids for protein synthesis. A smaller fraction surprisingly localizes to the Z-disc, where OGDH may locally provide amino acids for protein synthesis or may ‘moonlight’ in a different function, e.g. a structural role at the Z-disc.

Based on the currently available data, we propose that OGDH has dual roles in myofibril growth and Z-disc assembly: mitochondrial OGDH contributes to global amino acid production required for myofibril growth, whereas Z-disc OGDH is necessary to provide amino acids at the Z-disc for local protein synthesis that results in proper Z-disc growth and assembly. We can exclude a role for mitochondrial ATP production, because two different RNAi lines depleting cytochrome c oxidase do not affect myofibril growth or assembly (Fig. 6B). Metabolomics confirms, as expected, that amino acid levels are severely misregulated in OGDH mutants, which could therefore cause the observed phenotypes (Fig. 7). When OGDH function is impaired, many amino acids appear to be affected, some accumulate and others have highly reduced levels. The most affected amino acid is histidine. Histidine availability is linked to ribosome biogenesis. In fast-growing epithelial cells, high levels of histidine are required for ribosome biogenesis (Froldi et al., 2019). In addition, the loss of SLC15, a histidine amino acid transporter, correlates with low cytoplasmic histidine levels and with disruption of the mTOR pathway, which in turn controls ribosome biogenesis (Iadevaia et al., 2014; Kobayashi et al., 2014). Although we have not explored the requirements of specific amino acids, we speculate that low levels of histidine, like the ones in OGDH-HM muscles, are sufficient to reduce ribosome biogenesis. Many myofibrils have a smaller diameter in OGDH and other TCA cycle mutants, which is consistent with a global downregulation of histidine that results in reduced protein synthesis. However, the main OGDH phenotype cannot be explained by global downregulation of amino acids, because OGDH specifically affects Z-disc growth and assembly, but not M-line growth (Fig. 2). In contrast, global shutdown of protein synthesis disrupts only myofibril growth: myofibrils stop growing whenever protein synthesis is shut down, but sarcomeres look normal except for their smaller diameter (Fig. 8). We believe that a local production of amino acids at the Z-disc is more likely than a ‘moonlighting’ function of OGDH for three reasons: first, ribosomes are also enriched at the Z-disc, and their Z-disc localization depends on OGDH (Fig. 8); second, all three components of the OGDH complex and other TCA cycle components are found at the Z-disc, which would not be necessary if OGDH-E1 ‘moonlighted’ as a structural protein; and third, it appears unlikely that recruitment and precise localization to the Z-disc of the OGDH complex evolves without any associated function in amino acid production. To better dissect these contributions in the future will require OGDH mutants that cleanly disrupt enzymatic functions versus localization. Such mutants are currently unavailable, e.g. the OGDH-BM mutant identifies the area required for Z-disc localization (Figs 3 and 4), but may also affect enzymatic functions because it lies within the transketolase domain (Fig. 3B). An additional complication is that genetic mutants in these essential metabolic components are typically embryonic lethal and can therefore not be analyzed in adult muscles.

In conclusion, we observe a global alteration of amino acid homeostasis in OGDH mutants and specific localization of the OGDH complex to Z-discs, which aligns well with perturbed myofibril growth and specific Z-disc defects. This suggests important functions of the OGDH complex that go beyond its classical role in mitochondria.

## MATERIALS AND METHODS

### Experimental model

As a model organism, we used *Drosophila melanogaster.* Flies were raised at 25°C on standard cornmeal glucose media. A comprehensive list of all strains used and generated can be found in Table S2. Briefly, we used *Saccharomyces cerevisiae* for the yeast two-hybrid assays. The UAS/Gal4 system was used for transgene expression. The *Act88F-Gal4* transgene was used to direct expression in the indirect flight muscles (Bryantsev et al., 2012). *OGDH-TRiP.HMS00554* expresses RNAi against *OGDH* under UAS control (BDSC:33686). *OGDH^MI06026-GFSTF.1^* is a GFP trap allele of *OGDH* (BDSC:59416). *UAS-LacZ* (BDSC:3356) was used to express LacZ. *OGDH-GD.50393* expresses RNAi against *OGDH* under UAS control (VDRC:50393). Zasp52^MI02988-mCherry^, is a mCherry trap allele of *Zasp52* (Xiao et al., 2017). *Zasp52-GFP Zasp52^ZCL423^* is a GFP trap allele of *Zasp52* (BDSC:58790). *Zasp66-GFP Zasp66^ZCL0663^* is a GFP trap allele of *Zasp66* (BDSC:6824). Obscurin-GFP is a fosmid duplication from the fTRG library of the obscurin locus with a GFP at the C terminus. Sls-GFP is a GFP trap allele of *Sls* (Orfanos et al., 2015). *Zasp52^MI02988^* (BDSC_41034) and *Zasp52^MI00979^* (BDSC:33099) are MIMIC-based alleles of *Zasp52* that introduce early stop codons. They have been described previously (González-Morales et al., 2019b). *DLD-GFP* is a fosmid duplication from the fTRG library of the *DLD* locus with a GFP at the C terminus (VDRC:318906). *DLST-GFP* is a fosmid duplication from the fTRG library of the *DLST* locus. *UAS-RA.CS2* expresses a cold-sensitive ricin toxin under UAS control (BDSC:538624). *UAS-RpS5a-Venus* was used as Ribosome-GFP and is a gift from Paul Lasko (McGill University, Quebec, Canada) (Kong et al., 2019). The following RNAi strains targeting different TCA cycle enzymes or cytochrome c oxidase were used: mAcon1-KK103809 (VDRC:103809), Idh3b-KK102960 (VDRC:102960), Idh3b-GD6219 (VDRC:14443), Idh3a-PGD5222 (VDRC:41191), Idh3a-GD5222 (VDRC:41192), Idh3a-GD16641 (VDRC:50828), Idh3a-KK107912 (VDRC:107912), CG5028-GD6271 (VDRC:52043), CG5028-KK102781 (VDRC:102781), Fum1-KK108008 (VDRC:108008), Fum1-HMC03334 (BDSC:51779), Cox5A-HMJ22367 (BDSC:58282), Cox5A-JF02700 (BDSC:27548), DLST-HMC03051 (BDSC:50650), DLST-KK109081 (VDRC:109081), DLD-KK102614 (VDRC:102614), kdn-KK107737 (VDRC:107737), mdh2-KK109040 (VDRC:109040), mdh1-KK108844 (VDRC:108844), skap-KK101171 (VDRC:101171), Scsα1-KK102542 (VDRC:102542), ScsβG-KK109063 (VDRC:109063), UAS-Idh-Flag (BDSC:56202) and Acon1^CC00758^ (BDSC:51542).

### Evolutionary rate covariation

We obtained the ERC values from a previously characterized project (Findlay et al., 2014). We used Zasp66, Zasp52 and actinin as baits. This set contains pair-wise ERC values from 11,100 proteins calculated form multiple alignments of 12 *Drosophila* species. We then selected all the proteins with ERC values above 0.5 when compared with the bait proteins. We retrieved 94 proteins for Zasp66, 32 for Actinin and 25 for Zasp52. We used R to plot the values from the subset as a heat map and selected the proteins common to at least two of the bait proteins. Sixteen proteins were selected. From these, only OGDH and TER94 localized to the Z-disc.

### Confocal microscopy imaging of flight muscles

The muscles were prepared for confocal imaging as described previously (Xiao et al., 2017). Briefly, the thoraces were dissected in half and incubated overnight at −20°C in relaxing-glycerol solution [20 mM sodium phosphate (pH 7.2), 2 mM MgCl_2_, 2 mM EGTA, 5 mM DTT, 0.5% Triton X-100 and 50% glycerol]. We then fixed the muscles in 4% paraformaldehyde and dissected them. For visualizing actin filaments, we used 488-phalloidin or 555-phalloidin (1:1000; Cytoskeleton) in PBS. Finally, we mounted the samples in Mowiol 4-88 mounting media (Sigma, 9002-89-5). All images were acquired using a 63×1.4 NA HC Plan Apochromat oil objective on a Leica SP8 confocal microscope. We used more than 10 flies for each experiment and randomly picked the muscle area to image. Control and experimental samples were prepared and imaged simultaneously, and imaged with comparable parameters. We used muscles from very young flies – 1-2 days old.

### Bimolecular fluorescence complementation assay

BiFC assays were carried out as previously described (Marescal et al., 2020). The UAS-OGDH-NYFP construct was made using Gateway cloning using the OGDH-GEO09867 donor vector that contains the PA isoform as a donor and pBIDUAS-GV, pUAST-RfB-myc-NYFP as destination vector (Gohl et al., 2010). To make the UAS-OGDH-BM-NYFP construct, we first deleted the coding sequence for amino acids 741-769 in the OGDH-GEO09867 donor vector. The resulting vector was then transferred to pUAST-RfB-myc-NYFP using Gateway cloning. The resulting vectors were sequence verified and then inserted into the ZH-58A attp landing site. The UAS-Zasp52-PK-CYFP and the control lines have been described previously (Gohl et al., 2010; González-Morales, 2019b). At least 10 samples were used for each condition. We normalized the data to the basal noise levels and made plots in R software.

### Tissue-specific CRISPR mutants

Tissue-specific CRISPR disruption works by expressing the Cas9 endonuclease in a specific tissue, using the UAS/Gal4 system together with a gene targeting gRNA expressed ubiquitously. The tissue containing the Cas9 protein generates small insertion or deletion mutations in the gene targeted by the gRNA (Port et al., 2014). To express the Cas9 protein in the IFM, we used Act88F-Gal4 with UAS-Cas9.P2 (BDSC:58986). As gRNA constructs, we used TKO.GS03432 targeting DLST (CG5214), TKO.GS00548 targeting DLD (CG7430), and TKO.GS00550 targeting OGDH (Nc73EF). The muscle defects were observed in 1- to 2-day-old flies.

### Construction of DLST-GFP line

The fosmid carrying the GFP-tagged version of DLST (SourceBioscience: CBGtg9060A03104D) is part of the Flyfos library, a collection of fosmids that contain C-terminally GFP-tagged versions of genes at their genomic locations (Sarov et al., 2016). These constructs are then introduced into the fly genome by site-directed integration using the PhiC31 integrase (Bischof et al., 2007). We used P[CaryP]attP40 as the landing site for the DLST-GFP fosmid. Genome ProLab did the microinjections and Px3-RFP was used to screen for successful transformants.

### Precise genome engineering at the OGDH locus

To precisely modify the OGDH locus, we used the recombination-mediated cassette exchange method using *OGDH^MI06026^*, which carries a MiMIC transposon between exons 5 and 6 (Venken et al., 2011). The rationale was to replace the MiMIC transposon with wild-type or mutant versions of the OGDH gene, starting with the sequence where *OGDH^MI06026^* is inserted. In all cases, we added a C-terminal tag consisting of 6XHis and mCherry. First, we gene-synthesized the wild-type replacement construct and then mutagenized that construct using site-directed mutagenesis in bacteria. Gene synthesis and mutagenesis were carried out by Genscript. We created *OGDH^Δ741-769^* (*OGDH-BM*) by deleting residues 741 to 769. Residue numbering is in accordance with the OGDH-PA isoform. All constructs were then introduced into *OGDH^MI06026^* as a landing site. GenetiVision carried out the microinjections and initial confirmation of the mutants.

### Yeast two-hybrid assays

Yeast two-hybrid assays were carried out as described previously (González-Morales et al., 2019b).

### GC-MS sample preparation and metabolite measurements

The thoraces were dissected then flash frozen and crushed by mortar and pestle on liquid nitrogen (40 thoraces per sample). Frozen tissue powder was placed in pre-chilled Eppendorf brand tubes to which 1 ml of 80% methanol in water was added along with four 2.8 mm ceramic beads. Samples were subjected to 45 s of bead beating at 50 Hz (SpeedMill Plus homogenizer) four times. Samples were kept on ice between bead-beating sessions. Samples were then centrifuged at 1°C for 10 min at 21,130 ***g***. Supernatants were transferred to fresh pre-chilled tubes containing 1 µl of 800 ng/µl 2H27-Myristic in pyridine. The protein concentration of the pellets was estimated and used for normalization. Samples were then dried by vacuum centrifugation operating at a sample temperature of −4°C (LabConco).

After drying, samples were subjected to a two-step derivatization: First, the samples were resuspended in 30 µl of 10 mg/ml methoxyamine:HCl in anhydrous pyridine (MOX). They were sonicated and vortexed for 15 s three times then centrifuged for 3 min at room temperature at 21,130 ***g***. Incubation for methoximation was 30 min at room temperature. The samples were then centrifuged for 2 min at 21,130 ***g*** and the supernatants were transferred to GC-MS sample vials containing 250 µl glass inserts pre-filled with 70 µl of N-tert-butyldimethylsilyl-N-methyltrifluoroacetamide (MTBSTFA) and incubated at 70°C for 60 min.

An Agilent 5975C GC-MS equipped with a DB-5MS+DG (30 m×250 µm×0.25 µm) capillary column (Agilent J&W) was used for all GC-MS measurements, and data were collected by electron impact set at 70 eV both in scan (50-1000 *m*/*z*) and single ion monitoring modes. A volume of 1 ml of derivatized sample was injected in splitless mode with an inlet temperature set to 280°C, using helium as a carrier gas, and the flow rate was adjusted to 18 min for 2H27-myristic acid. The quadrupole was set at 150°C and the GC-MS interface at 285°C. The oven program for all metabolite analyses started at 60°C held for 1 min, then increased at a rate of 10°C/min until 320°C. Bake-out was at 320°C for 10 min. Sample data were acquired in scan mode (50-1000 *m*/*z*) or in single ion monitoring (SIM) with a 5 ms dwell time where the M-57 [M+•-C4H9•]+ fragment was used for quantitation (area under the curve) in both modes of data acquisition. Citrate and isocitrate used the *m*/*z* 459 ion for quantification as described previously (Mamer et al., 2013). The spectra and retention times of all metabolites reported were confirmed by methoxylamine–tert-butyldimethylsilylated authentic standards. For saturating metabolites, samples were diluted 1:25 with the same ratio of derivatization reagents and run in scan mode. Metabolite area under the curve was normalized to tissue weight.

### Transmission electron microscopy

Muscles samples from 1- to 2-day-old flies were prepared for transmission electron microscopy imaging as described previously with slight modifications (González-Morales et al., 2017). Briefly, the thoraces were dissected in half and were treated with 5 mM MOPS (pH 6.8), 150 mM KCl, 5 mM EGTA, 5 mM ATP and 1% Triton X-100 for 2 h at 4°C. Samples were then washed in rigor solution [5 mM MOPS (pH 6.8), 40 mM KCl, 5 mM EGTA, 5 mM MgCl2 and 5 mM NaN3] and fixed in 3% glutaraldehyde, 0.2% tannic acid in 20 mM MOPS (pH 6.8), 5 mM EGTA, 5 mM MgCl_2_ and 5 mM NaN3 for 2 h at 4°C. Images were acquired on a Tecnai 12 BioTwin 120 kV transmission electron microscope with an AMT XR80C CCD camera (FEI).

### Super-resolution dSTORM microscopy

Super-resolution imaging was carried out essentially as described previously (Szikora et al., 2020). Briefly, all the dSTORM images were captured under EPI illumination (Nikon CFI Apo 100×, NA=1.49) on a custom-made inverted microscope based on a Nikon Eclipse Ti-E frame. The laser (MPB Communication; 647 nm, Pmax=300 mW) intensity was controlled via an acousto-optic tunable filter (AOTF) set to 2-4 kW/cm^2^ on the sample plane. An additional laser (Nichia: 405 nm, Pmax=60 mW) was used for reactivation. Images were captured by an Andor iXon3 897 BV EMCCD digital camera (512×512 pixels with 16 μm pixel size). Frame stacks for dSTORM super-resolution imaging were captured at a reduced image size. A fluorescence filter set (Semrock, LF405/488/561/635-A-000) with an additional emission filter (AHF, 690/70 H Bandpass) was used to select and separate the excitation and emission lights in the microscope. During the measurements, the perfect focus system of the microscope was used to keep the sample in focus with a precision of <30 nm. Immediately before the measurement, the storage buffer of the sample was replaced with a GLOX switching buffer (van de Linde et al., 2011), and the sample was mounted onto a microscope slide. Typically, 20,000-50,000 frames were captured with an exposure time of 20 or 30 ms. The captured and stored image stacks were evaluated and analyzed with the rainSTORM localization software (Rees et al., 2013). Individual images of single molecules were fitted with a Gaussian point spread function and their center positions were associated with the position of the fluorescent molecule. Localizations were filtered via their intensity, precision and standard deviation values. Only localizations with precisions of <20 nm and standard deviation (σ) of 0.8≤σ≤1.0 were used to form the final image and for further analysis. Mechanical drift introduced by either the mechanical movement of the sample or thermal effects was analyzed and reduced using a correlation-based blind drift correction algorithm. Spatial coordinates of the localized events were stored, and the final super-resolved image was visualized with a pixel size of 10 nm. We used GFP-tagged lines to determine the nanoscopic localization of OGDH, DLD and DLST. Individual myofibrils were isolated from the IFM of anesthetized adult (∼24 h after eclosion) *Drosophila* as described previously (Burkart et al., 2007), with minor modifications. In brief, bisected hemithoraces were incubated in relaxing solution [100 mM NaCl, 20 mM NaPi (pH, 7.0), 5 mM MgCl_2_, 5 mM EGTA and 5 mM ATP] supplemented with 50% glycerol for 2 h at 4°C. Afterwards, the dorsal longitudinal muscles were isolated from the hemithoraces and dissociated by gently pipetting them in an Eppendorf tube in the presence of 0.5% Triton X-100. Dissociated myofibrils were centrifuged at 12,300 ***g*** for 2 min. Myofibrils were washed and centrifuged two more times in relaxing solution. Myofibrils were resuspended in a relaxing solution, and 20 µl of the sample was dropped on a glass coverslip and fixed with 4% paraformaldehyde (Alfa Aesar) in relaxing solution for 15 min. After washing three times in relaxing solution, the samples were blocked in blocking solution [5% goat serum (Sigma) and 0.1% Triton X-100 in relaxing solution] for 30 min in a humidity chamber. To detect OGDH, DLD and DLST-GFP, an anti-GFP antibody (1:1000; Abcam; ab13970) was applied overnight at 4°C in a blocking solution. After washing, goat anti-chicken secondary antibody coupled to AlexaFluor 647 (1:600; Invitrogen; A21449) was applied for 2 h at room temperature. F-actin was labeled with AlexaFluor 488-phalloidin (1:200; Thermo Fisher Scientific; A12379). The samples were thoroughly washed and stored in PBS before imaging. Experimental spatial resolution and localization precision were determined by the Fourier Ring Correlation and the Nearest Neighbor approaches (Endesfelder et al., 2014; Nieuwenhuizen et al., 2013). Drift-corrected measurement data generated by rainSTORM were first preconditioned and then evaluated by the FIRE and Coordinate Based Localization Precision Estimator codes. Spatial resolution of 52.8±6.3 nm and localization precision of 10.5±1.5 nm were achieved by the evaluation of ten randomly selected datasets. The values did not show any correlation with the samples, they were instead specified by the dSTORM microscope system and the data acquisition process. Based on these experimental results, localizations with theoretical (Thompson) localization precisions of less than 20 nm and a standard deviation (σ) of 0.8≤σ≤1.0 were used to form the final image and for further analysis.

### Ribosome block

To block protein synthesis, we used UAS-RA.cs2, a cold-sensitive version of the ricin-A toxin subunit. Ricin-A inactivates ribosomes by the specific depurination of the 28S rRNA (Moffat et al., 1992). Ricin-A-TS is a temperature-sensitive allele that is active at 30°C but not at 20°C. We raised Act88F-Gal4 UAS-Ricin-A-TS flies at 20°C then transferred them to a 30°C incubator for 48 h, and then back into 20°C. The muscles were analyzed 2 days after emergence.

## Supplementary Material

10.1242/joces.260717_sup1 Supplementary information

## References

[JCS260717C1] Ahmed, R. E., Tokuyama, T., Anzai, T., Chanthra, N. and Uosaki, H. (2022). Sarcomere maturation: function acquisition, molecular mechanism, and interplay with other organelles. *Philos. Trans. R. Soc. Lond. B Biol. Sci.* 377, 20210325. 10.1098/rstb.2021.0325 36189811 PMC9527934

[JCS260717C2] Avellaneda, J., Rodier, C., Daian, F., Brouilly, N., Rival, T., Luis, N. M. and Schnorrer, F. (2021). Myofibril and mitochondria morphogenesis are coordinated by a mechanical feedback mechanism in muscle. *Nat. Commun.* 12, 2091. 10.1038/s41467-021-22058-7 33828099 PMC8027795

[JCS260717C3] Bischof, J., Maeda, R. K., Hediger, M., Karch, F. and Basler, K. (2007). An optimized transgenesis system for Drosophila using germ-line-specific phiC31 integrases. *Proc. Natl. Acad. Sci. USA* 104, 3312-3317. 10.1073/pnas.0611511104 17360644 PMC1805588

[JCS260717C4] Bryantsev, A. L., Duong, S., Brunetti, T. M., Chechenova, M. B., Lovato, T. L., Nelson, C., Cripps, R. M., Uhl, J. D. and Gebelein, B. (2012). Extradenticle and homothorax control adult muscle fiber identity in Drosophila. *Dev. Cell* 23, 664-673. 10.1016/j.devcel.2012.08.004 22975331 PMC3575643

[JCS260717C5] Burkart, C., Qiu, F., Brendel, S., Benes, V., Haag, P., Labeit, S. and Bullard, B. (2007). Modular proteins from the Drosophila sallimus (sls) gene and their expression in muscles with different extensibility. *J. Mol. Biol.* 367, 953-969. 10.1016/j.jmb.2007.01.059 17316686

[JCS260717C6] Chen, C.-L., Hu, Y., Udeshi, N. D., Lau, T. Y., Wirtz-Peitz, F., He, L., Ting, A. Y. and Carr, S., A. and Perrimon, N. (2015). Proteomic mapping in live Drosophila tissues using an engineered ascorbate peroxidase. *Proc. Natl. Acad. Sci. USA* 112, 12093-12098. 10.1073/pnas.1515623112 26362788 PMC4593093

[JCS260717C7] Chu, W. C. and Hayashi, S. (2021). Mechano-chemical enforcement of tendon apical ECM into nano-filaments during Drosophila flight muscle development. *Curr. Biol.* 31, 1366-1378 e7. 10.1016/j.cub.2021.01.010 33545042

[JCS260717C8] Clark, N. L. and Aquadro, C. F. (2010). A novel method to detect proteins evolving at correlated rates: identifying new functional relationships between coevolving proteins. *Mol. Biol. Evol.* 27, 1152-1161. 10.1093/molbev/msp324 20044587 PMC2877527

[JCS260717C9] Demontis, F. and Perrimon, N. (2009). Integration of Insulin receptor/Foxo signaling and dMyc activity during muscle growth regulates body size in Drosophila. *Development* 136, 983-993. 10.1242/dev.027466 19211682 PMC2727562

[JCS260717C10] Denes, L. T., Kelley, C. P. and Wang, E. T. (2021). Microtubule-based transport is essential to distribute RNA and nascent protein in skeletal muscle. *Nat. Commun.* 12, 6079. 10.1038/s41467-021-26383-9 34707124 PMC8551216

[JCS260717C11] Destefanis, F., Manara, V. and Bellosta, P. (2020). Myc as a regulator of ribosome biogenesis and cell competition: a link to cancer. *Int. J. Mol. Sci.* 21, 4037. 10.3390/ijms21114037 32516899 PMC7312820

[JCS260717C12] Dos Remedios, C. and Gilmour, D. (2017). An historical perspective of the discovery of titin filaments. *Biophys. Rev.* 9, 179-188. 10.1007/s12551-017-0269-3 28656582 PMC5498331

[JCS260717C13] Endesfelder, U., Malkusch, S., Fricke, F. and Heilemann, M. (2014). A simple method to estimate the average localization precision of a single-molecule localization microscopy experiment. *Histochem. Cell Biol.* 141, 629-638. 10.1007/s00418-014-1192-3 24522395

[JCS260717C14] Fernandes, I. and Schöck, F. (2014). The nebulin repeat protein Lasp regulates I-band architecture and filament spacing in myofibrils. *J. Cell Biol.* 206, 559-572. 10.1083/jcb.201401094 25113030 PMC4137052

[JCS260717C15] Findlay, G. D., Sitnik, J. L., Wang, W., Aquadro, C. F., Clark, N. L. and Wolfner, M. F. (2014). Evolutionary rate covariation identifies new members of a protein network required for Drosophila melanogaster female post-mating responses. *PLoS Genet.* 10, e1004108. 10.1371/journal.pgen.1004108 24453993 PMC3894160

[JCS260717C16] Froldi, F., Pachnis, P., Szuperak, M., Costas, O., Fernando, T., Gould, A. P. and Cheng, L. Y. (2019). Histidine is selectively required for the growth of Myc-dependent dedifferentiation tumours in the Drosophila CNS. *EMBO J.* 38, e99895. 10.15252/embj.201899895 30804004 PMC6443203

[JCS260717C17] Gohl, C., Banovic, D., Grevelhorster, A. and Bogdan, S. (2010). WAVE forms hetero- and homo-oligomeric complexes at integrin junctions in Drosophila visualized by bimolecular fluorescence complementation. *J. Biol. Chem.* 285, 40171-40179. 10.1074/jbc.M110.139337 20937809 PMC3000999

[JCS260717C18] González-Morales, N., Marsh, T. W., Katzemich, A., Marescal, O., Xiao, Y. S. and Schöck, F. (2019a). Different evolutionary trajectories of two insect-specific paralogous proteins involved in stabilizing muscle myofibrils. *Genetics* 212, 743-755. 10.1534/genetics.119.302217 31123042 PMC6614898

[JCS260717C19] González-Morales, N., Xiao, Y. S., Schilling, M. A., Marescal, O., Liao, K. A. and Schöck, F. (2019b). Myofibril diameter is set by a finely tuned mechanism of protein oligomerization in Drosophila. *Elife,* 8, 50496. 10.7554/eLife.50496 PMC6910826 31746737

[JCS260717C20] Gruntenko, N. E., Kochetov, A. V., Makarova, K. S., Mishin, V. P., Lukasheva, V. V., Ptitsyn, A. A. and Kokoza, V. A. (1998). [Gene Nc73EF of Drosophila melanogaster encodes a protein highly homologous to E1 subunit of human 2-oxoglutarate dehydrogenase]. *Genetika* 34, 32-37. 9532450

[JCS260717C21] Gunage, R. D., Dhanyasi, N., Reichert, H. and Vijayraghavan, K. (2017). Drosophila adult muscle development and regeneration. *Semin. Cell Dev. Biol.* 72, 56-66. 10.1016/j.semcdb.2017.11.017 29146144

[JCS260717C22] Iadevaia, V., Liu, R. and Proud, C. G. (2014). mTORC1 signaling controls multiple steps in ribosome biogenesis. *Semin. Cell Dev. Biol.* 36, 113-120. 10.1016/j.semcdb.2014.08.004 25148809

[JCS260717C23] Katzemich, A., Kreiskother, N., Alexandrovich, A., Elliott, C., Schöck, F., Leonard, K., Sparrow, J. and Bullard, B. (2012). The function of the M-line protein obscurin in controlling the symmetry of the sarcomere in the flight muscle of Drosophila. *J. Cell Sci.* 125, 3367-3379. 22467859 10.1242/jcs.097345 PMC3516378

[JCS260717C24] Katzemich, A., Liao, K. A., Czerniecki, S. and Schöck, F. (2013). Alp/Enigma family proteins cooperate in Z-disc formation and myofibril assembly. *PLoS Genet.* 9, e1003342. 10.1371/journal.pgen.1003342 23505387 PMC3591300

[JCS260717C25] Kaya-Copur, A., Marchiano, F., Hein, M. Y., Alpern, D., Russeil, J., Luis, N. M., Schnorrer, F., Hosaka, T., Goto, M. and Kato, N. (2021). The Hippo pathway controls myofibril assembly and muscle fiber growth by regulating sarcomeric gene expression. *Elife* 10, e63726. 10.7554/eLife.63726 33404503 PMC7815313

[JCS260717C26] Kobayashi, T., Shimabukuro-Demoto, S., Yoshida-Sugitani, R., Furuyama-Tanaka, K., Karyu, H., Sugiura, Y., Shimizu, Y., Hosaka, T., Goto, M., Kato, N. et al. (2014). The histidine transporter SLC15A4 coordinates mTOR-dependent inflammatory responses and pathogenic antibody production. *Immunity* 41, 375-388. 10.1016/j.immuni.2014.08.011 25238095

[JCS260717C27] Kong, J., Han, H., Bergalet, J., Bouvrette, L. P. B., Hernandez, G., Moon, N. S., Vali, H., Lécuyer, É. and Lasko, P. (2019). A ribosomal protein S5 isoform is essential for oogenesis and interacts with distinct RNAs in Drosophila melanogaster. *Sci. Rep.* 9, 13779. 10.1038/s41598-019-50357-z 31551467 PMC6760144

[JCS260717C28] Kyrilis, F. L., Semchonok, D. A., Skalidis, I., Tuting, C., Hamdi, F., O'reilly, F. J. and Kastritis, P. L. (2021). Integrative structure of a 10-megadalton eukaryotic pyruvate dehydrogenase complex from native cell extracts. *Cell Rep.* 34, 108727. 10.1016/j.celrep.2021.108727 33567276

[JCS260717C29] Larkin, A., Marygold, S. J., Antonazzo, G., Attrill, H., Dos Santos, G., Garapati, P. V., Goodman, J. L., Gramates, L. S., Millburn, G., Strelets, V. B. et al. (2021). FlyBase: updates to the Drosophila melanogaster knowledge base. *Nucleic Acids Res.* 49, D899-D907. 10.1093/nar/gkaa1026 33219682 PMC7779046

[JCS260717C30] Lemke, S. B. and Schnorrer, F. (2017). Mechanical forces during muscle development. *Mech. Dev.* 144 (Pt A), 92-101. 10.1016/j.mod.2016.11.003 27913119

[JCS260717C31] Liao, K. A., González-Morales, N. and Schöck, F. (2016). Zasp52, a core Z-disc protein in Drosophila indirect flight muscles, interacts with alpha-actinin via an extended PDZ domain. *PLoS Genet.* 12, e1006400. 10.1371/journal.pgen.1006400 27783625 PMC5081203

[JCS260717C32] Loison, O., Weitkunat, M., Kaya-Copur, A., Nascimento Alves, C., Matzat, T., Spletter, M. L., Luschnig, S., Brasselet, S., Lenne, P. F. and Schnorrer, F. (2018). Polarization-resolved microscopy reveals a muscle myosin motor-independent mechanism of molecular actin ordering during sarcomere maturation. *PLoS Biol.* 16, e2004718. 10.1371/journal.pbio.2004718 29702642 PMC5955565

[JCS260717C33] Luis, N. M. and Schnorrer, F. (2021). Mechanobiology of muscle and myofibril morphogenesis. *Cells Dev.* 168, 203760. 10.1016/j.cdev.2021.203760 34863916

[JCS260717C70] Mamer, O., Gravel, S. P., Choinière, L., Chénard, V., St-Pierre, J. and Avizonis, D. (2013). The complete targeted profile of the organic acid intermediates of the citric acid cycle using a single stable isotope dilution analysis, sodium borodeuteride reduction and selected ion monitoring GC/MS. *Metabolomics* 9, 1019-1030. 10.1007/s11306-013-0521-1 24348278 PMC3855487

[JCS260717C34] Mandal, S., Guptan, P., Owusu-Ansah, E. and Banerjee, U. (2005). Mitochondrial regulation of cell cycle progression during development as revealed by the tenured mutation in Drosophila. *Dev. Cell* 9, 843-854. 10.1016/j.devcel.2005.11.006 16326395

[JCS260717C35] Marescal, O., Schöck, F. and González-Morales, N. (2020). Bimolecular fluorescence complementation (BiFC) for studying sarcomeric protein interactions in Drosophila. *Bio Protoc.* 10, e3569. 10.21769/BioProtoc.3569 PMC7842562 33659539

[JCS260717C36] Martinez-Reyes, I. and Chandel, N. S. (2020). Mitochondrial TCA cycle metabolites control physiology and disease. *Nat. Commun.* 11, 102. 10.1038/s41467-019-13668-3 31900386 PMC6941980

[JCS260717C37] Moffat, K. G., Gould, J. H., Smith, H. K. and O'kane, C. J. (1992). Inducible cell ablation in Drosophila by cold-sensitive ricin A chain. *Development* 114, 681-687. 10.1242/dev.114.3.681 1618135

[JCS260717C38] Molnar, I., Migh, E., Szikora, S., Kalmar, T., Vegh, A. G., Deak, F., Mihaly, J., Bugyi, B., Orfanos, Z., Kovács, J. et al. (2014). DAAM is required for thin filament formation and Sarcomerogenesis during muscle development in Drosophila. *PLoS Genet.* 10, e1004166. 10.1371/journal.pgen.1004166 24586196 PMC3937221

[JCS260717C40] Nieuwenhuizen, R. P., Lidke, K. A., Bates, M., Puig, D. L., Grunwald, D., Stallinga, S. and Rieger, B. (2013). Measuring image resolution in optical nanoscopy. *Nat. Methods* 10, 557-562. 10.1038/nmeth.2448 23624665 PMC4149789

[JCS260717C41] Nikonova, E., Kao, S. Y. and Spletter, M. L. (2020). Contributions of alternative splicing to muscle type development and function. *Semin. Cell Dev. Biol.* 104, 65-80. 10.1016/j.semcdb.2020.02.003 32070639

[JCS260717C42] Orfanos, Z., Leonard, K., Elliott, C., Katzemich, A., Bullard, B. and Sparrow, J. (2015). Sallimus and the dynamics of sarcomere assembly in Drosophila flight muscles. *J. Mol. Biol.* 427, 2151-2158. 10.1016/j.jmb.2015.04.003 25868382

[JCS260717C43] Owen, O. E., Kalhan, S. C. and Hanson, R. W. (2002). The key role of anaplerosis and cataplerosis for citric acid cycle function. *J. Biol. Chem.* 277, 30409-30412. 10.1074/jbc.R200006200 12087111

[JCS260717C44] Port, F., Chen, H. M., Lee, T. and Bullock, S. L. (2014). Optimized CRISPR/Cas tools for efficient germline and somatic genome engineering in Drosophila. *Proc. Natl. Acad. Sci. USA* 111, E2967-E2976. 10.1073/pnas.1405500111 25002478 PMC4115528

[JCS260717C46] Raza, Q., Choi, J. Y., Li, Y., O'dowd, R. M., Watkins, S. C., Chikina, M., Kwiatkowski, A. V., Clark, N. L. and Kwiatkowski, A. V. (2019). Evolutionary rate covariation analysis of E-cadherin identifies Raskol as a regulator of cell adhesion and actin dynamics in Drosophila. *PLoS Genet.* 15, e1007720. 10.1371/journal.pgen.1007720 30763317 PMC6375579

[JCS260717C47] Reedy, M. C. and Beall, C. (1993). Ultrastructure of developing flight muscle in Drosophila. I. Assembly of myofibrils. *Dev. Biol.* 160, 443-465. 10.1006/dbio.1993.1320 8253277

[JCS260717C48] Rees, E. J., Erdelyi, M., Schierle, G. S. K., Knight, A. and Kaminski, C. F. (2013). Elements of image processing in localization microscopy. *J. Opt.* 15, Artn 094012. 10.1088/2040-8978/15/9/094012

[JCS260717C50] Rudolph, F., Fink, C., Huttemeister, J., Kirchner, M., Radke, M. H., Lopez Carballo, J., Gotthardt, M., Kohl, T., Lehnart, S. E., Mertins, P. et al. (2020). Deconstructing sarcomeric structure-function relations in titin-BioID knock-in mice. *Nat. Commun.* 11, 3133. 10.1038/s41467-020-16929-8 32561764 PMC7305127

[JCS260717C51] Sarov, M., Barz, C., Jambor, H., Hein, M. Y., Schmied, C., Suchold, D., Schnorrer, F., Stender, B., Janosch, S., K J, V. V. et al. (2016). A genome-wide resource for the analysis of protein localisation in Drosophila. *Elife* 5, e12068. 10.7554/eLife.12068 26896675 PMC4805545

[JCS260717C53] Shwartz, A., Dhanyasi, N., Schejter, E. D. and Shilo, B.-Z. (2016). The Drosophila formin Fhos is a primary mediator of sarcomeric thin-filament array assembly. *Elife* 5, e16540. 10.7554/eLife.16540 27731794 PMC5061545

[JCS260717C54] Skalidis, I., Tuting, C. and Kastritis, P. L. (2020). Unstructured regions of large enzymatic complexes control the availability of metabolites with signaling functions. *Cell Commun. Signal.* 18, 136. 10.1186/s12964-020-00631-9 32843078 PMC7448341

[JCS260717C55] Spletter, M. L., Barz, C., Yeroslaviz, A., Zhang, X., Lemke, S. B., Bonnard, A., Bonnard, A., Brunner, E., Cardone, G., Basler, K. et al. (2018). A transcriptomics resource reveals a transcriptional transition during ordered sarcomere morphogenesis in flight muscle. *Elife* 7, e34058. 10.7554/eLife.34058 29846170 PMC6005683

[JCS260717C56] Sullivan, D. T., Macintyre, R., Fuda, N., Fiori, J., Barrilla, J. and Ramizel, L. (2003). Analysis of glycolytic enzyme co-localization in Drosophila flight muscle. *J. Exp. Biol.* 206, 2031-2038. 10.1242/jeb.00367 12756285

[JCS260717C57] Szikora, S., Gajdos, T., Novak, T., Farkas, D., Foldi, I., Lenart, P., Erdélyi, M. and Mihaly, J. (2020). Nanoscopy reveals the layered organization of the sarcomeric H-zone and I-band complexes. *J. Cell Biol.* 219, e201907026. 10.1083/jcb.201907026 31816054 PMC7039190

[JCS260717C58] Tretter, L. and Adam-Vizi, V. (2005). Alpha-ketoglutarate dehydrogenase: a target and generator of oxidative stress. *Philos. Trans. R. Soc. Lond. B Biol. Sci.* 360, 2335-2345. 10.1098/rstb.2005.1764 16321804 PMC1569585

[JCS260717C59] Tskhovrebova, L. and Trinick, J. (2017). Titin and nebulin in thick and thin filament length regulation. *Subcell Biochem.* 82, 285-318. 10.1007/978-3-319-49674-0_10 28101866

[JCS260717C60] Van De Linde, S., Loschberger, A., Klein, T., Heidbreder, M., Wolter, S., Heilemann, M. and Sauer, M. (2011). Direct stochastic optical reconstruction microscopy with standard fluorescent probes. *Nat. Protoc.* 6, 991-1009. 10.1038/nprot.2011.336 21720313

[JCS260717C61] Venken, K. J., Schulze, K. L., Haelterman, N. A., Pan, H., He, Y., Evans-Holm, M., Bellen, H. J., Levis, R. W., Spradling, A. C., Hoskins, R. A. et al. (2011). MiMIC: a highly versatile transposon insertion resource for engineering Drosophila melanogaster genes. *Nat. Methods* 8, 737-743. 10.1038/nmeth.1662 21985007 PMC3191940

[JCS260717C62] Weitkunat, M., Kaya-Copur, A., Grill, S. W. and Schnorrer, F. (2014). Tension and force-resistant attachment are essential for myofibrillogenesis in Drosophila flight muscle. *Curr. Biol.* 24, 705-716. 10.1016/j.cub.2014.02.032 24631244

[JCS260717C63] Weitkunat, M., Brasse, M., Bausch, A. R. and Schnorrer, F. (2017). Mechanical tension and spontaneous muscle twitching precede the formation of cross-striated muscle in vivo. *Development* 144, 1261-1272. 10.1242/dev.140723 28174246 PMC5399620

[JCS260717C64] Wojtas, K., Slepecky, N., Von Kalm, L. and Sullivan, D. (1997). Flight muscle function in Drosophila requires colocalization of glycolytic enzymes. *Mol. Biol. Cell* 8, 1665-1675. 10.1091/mbc.8.9.1665 9307964 PMC305727

[JCS260717C65] Xiao, Y. S., Schöck, F. and González-Morales, N. (2017). Rapid IFM dissection for visualizing fluorescently tagged sarcomeric proteins. *Bio Protoc.* 7, e2606. 10.21769/BioProtoc.2606 PMC5800882 29423427

[JCS260717C66] Yoon, W. H., Sandoval, H., Nagarkar-Jaiswal, S., Jaiswal, M., Yamamoto, S., Haelterman, N. A., Putluri, N., Putluri, V., Sreekumar, A., Tos, T. et al. (2017). Loss of Nardilysin, a Mitochondrial Co-chaperone for alpha-Ketoglutarate Dehydrogenase, Promotes mTORC1 activation and neurodegeneration. *Neuron* 93, 115-131. 10.1016/j.neuron.2016.11.038 28017472 PMC5242142

[JCS260717C67] Zappia, M. P. and Frolov, M. V. (2016). E2F function in muscle growth is necessary and sufficient for viability in Drosophila. *Nat. Commun.* 7, 10509. 10.1038/ncomms10509 26823289 PMC4740182

[JCS260717C68] Zappia, M. P., Rogers, A., Islam, A. and Frolov, M. V. (2019). Rbf activates the myogenic transcriptional program to promote skeletal muscle differentiation. *Cell Rep.* 26, 702-719.e6. 10.1016/j.celrep.2018.12.080 30650361 PMC6344057

