# Supplementary material for: The oxoglutarate dehydrogenase complex is involved in myofibril growth and Z-disc assembly in Drosophila
Source: J Cell Sci. 2023 Jun 30;136(13):jcs260717. doi: 10.1242/jcs.260717 (PMC10323237; doi:10.1242/jcs.260717)
Supplement: Supplementary information [file joces-136-260717-s1.pdf]

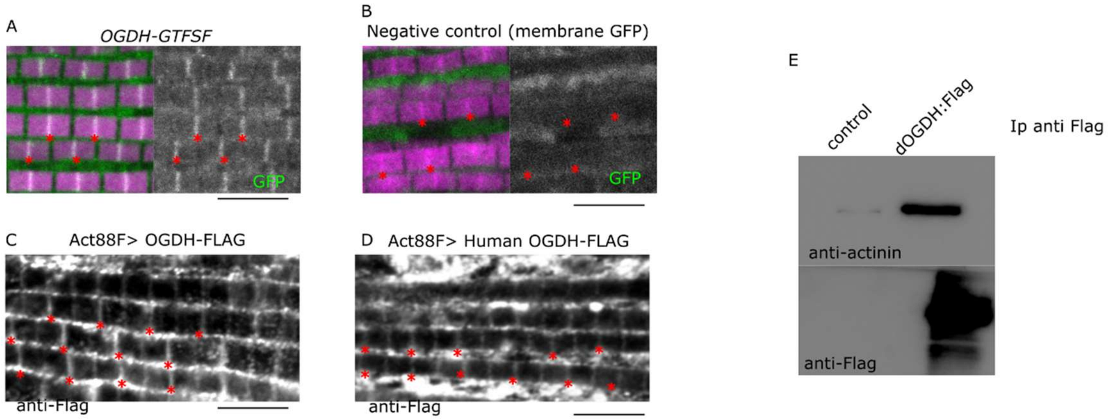

Fig. S1. Additional evidence of OGDH-E1 localization at the Z-disc. (A-B) OGDH-GFP has a clear localization signal (A), but a membrane GFP control does not (B). (C-D) Flag-tagged OGDH localizes to the Z-disc. Overexpression of *Drosophila* OGDH or Human OGDH results in a faint but detectable Z-disc signal. (E) immunoprecipitation experiment from Thorax extracts. The Z-disc protein α-actinin precipitates with OGDH-Flag but not in the control without OGDH-Flag.

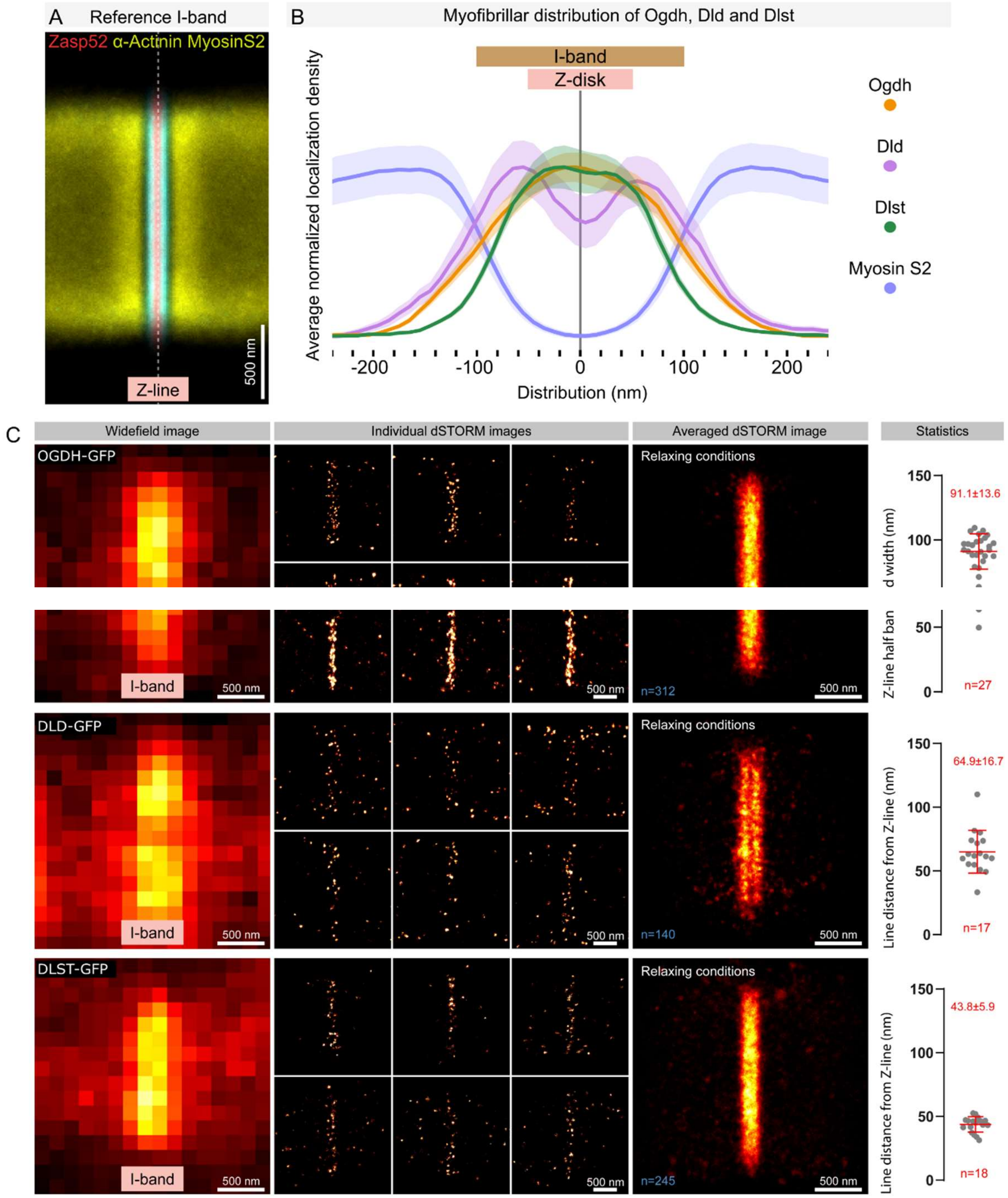

Fig. S2. Details of the dSTORM super-resolution imaging experiment.

(A) Reference I-band image showing the distribution patterns of Actinin, MyosinS2, and Zasp52 epitopes. (B) Longitudinal epitope average normalized localization epitope densities of the three OGDH complex subunits relative to the distribution of Myosin S2 epitope distribution pattern. (C) Examples of widefield images, individual dSTORM images, the average distribution image (as shown in Fig. 1C-E), and descriptive statistics of the distribution patterns for each subunit of the OGDH complex. The scale bar is 500 nm. The number of individual dSTORM images is shown in the average image. In the average dSTROM image, n represents the number of individual dSTORM images used to create the distribution average image. In the statistics, n represents the number of individual dSTORM average images.

Journal of Cell Science • Supplementary information

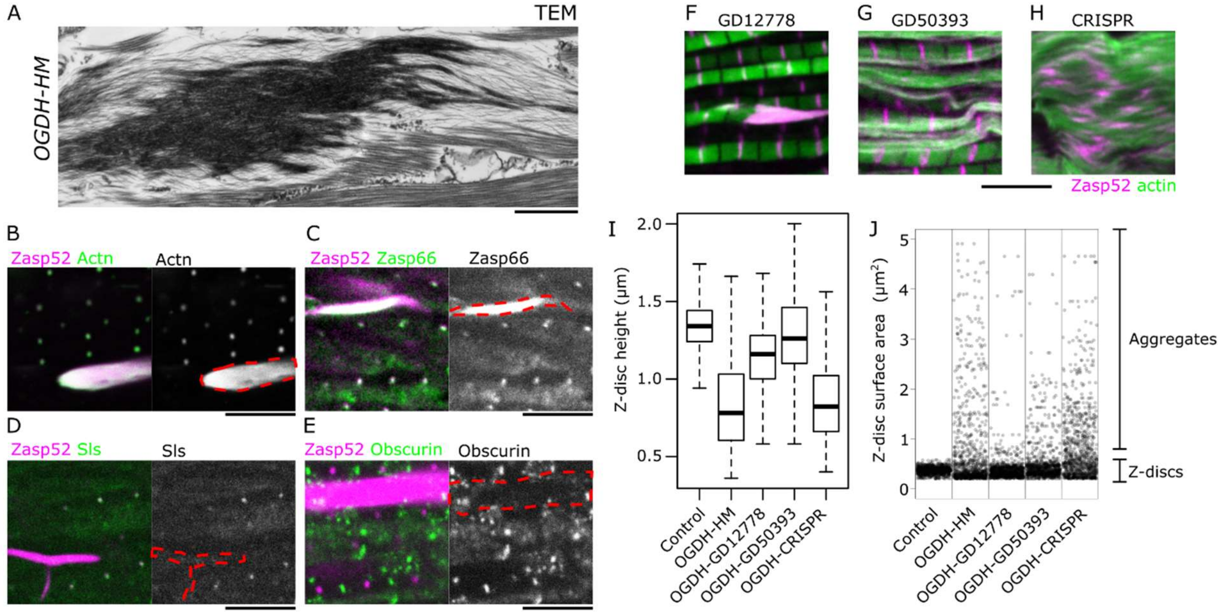

Fig. S3. Additional characterization of OGDH-HM myofibril phenotype. (A) TEM image of an aggregate formed in OGDH-HM muscles. Notice the actin filaments that connect to the aggregate. The scale bar is 1 µm. (B-E) Confocal images of OGDH-HM muscles with different sarcomere proteins labeled. In all cases, Zasp52 marks the Z-discs and the aggregates in magenta. Scale bars are 5 µm. (B) Actinin localizes to the small Z-discs and the aggregates. (C) Zasp66 localizes to the small Z-discs and the aggregates. (D) Sls/Titin localizes to the Z-discs but not the aggregates. (E) The M-line protein Obscurin does not localize to the Z-discs or the aggregates. (F-H) Confocal images of muscles in three alternative approaches to deplete OGDH. Actin filaments are shown in green and Zasp52 marks the Z-discs in magenta. (F) The RNAi GD12778 directed against OGDH results in small aggregates and myofibril defects. (G) The RNAi GD50393 directed against another region of OGDH results in myofibril disintegration. (H) The TKO CRISPR-based method directed against OGDH results in the complete disintegration of the myofibril structure. (I) Boxplot of Z-disc heights in different OGDH depleted conditions. (J) A plot of the surface area of Zasp52 positive particles. Small particles are Z-discs while large particles are aggregates. Notice the large number of aggregates in OGDH-depleted conditions. The flies are 1-2 days old.

Journal of Cell Science • Supplementary information

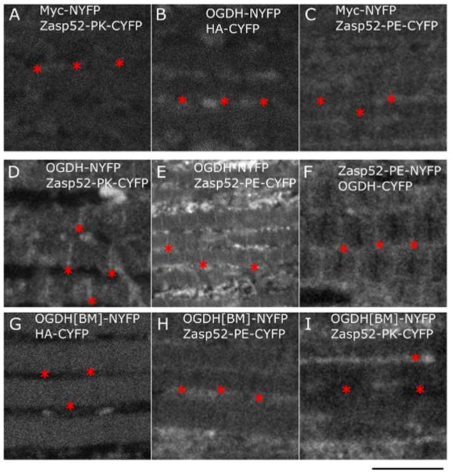

Fig. S4. Representative BiFC images from all conditions tested. (A-C) Control muscles show there is no binding between the C-terminal YFP or N-terminal YFP tags to either OGDH or Zasp52. Z-disc fluorescence is not observed. (D-E) Experimental groups testing binding between OGDH and Zasp52. A faint but reproducible signal is observed at the Z-disc. (F-G) Control muscles testing binding between the OGDH-BM mutant and Zasp52. Z-disc fluorescence is not observed. Zasp52-PK is a short splice variant with only one LIM domain and Zasp52-E is a long splice variant with 4 LIM domains. The scale bar is 5 µm. The red asterisks denote the position of the Z-discs inferred from F-actin co-staining.

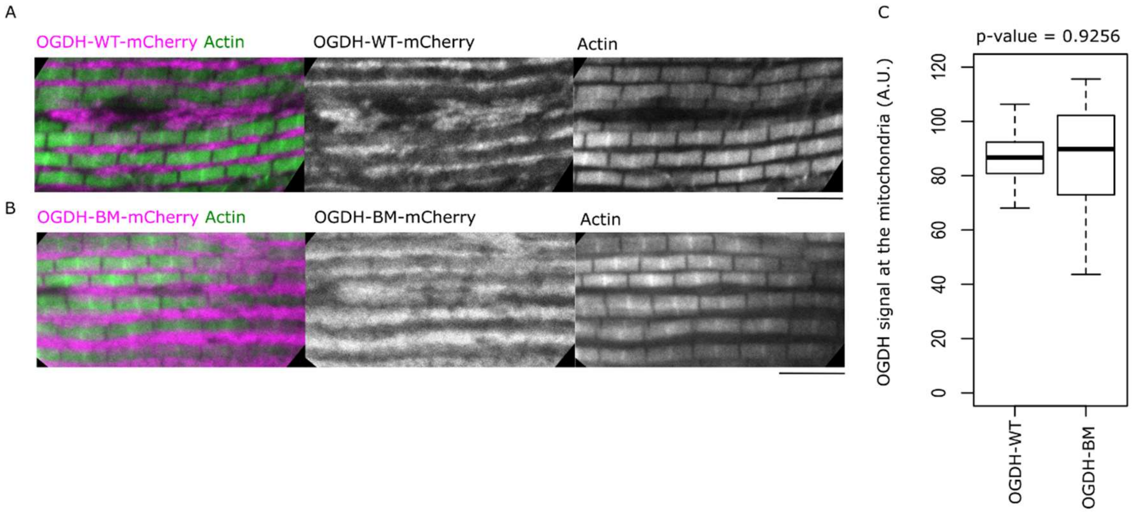

Fig. S5. OGDH-BM mutant does not affect mitochondria localization. (A-B) The wild type and the BM mutant versions of OGDH-mCherry show strong mitochondrial fluorescence. mCherry is shown in magenta and F-actin in green. The scale bar is 5 µm. (C) A plot of mCherry fluorescence intensity values in OGDH-WT-mCh and OGDH-BM-mCh. The p-value was calculated using Welch's two-sample t-test.

Journal of Cell Science • Supplementary information

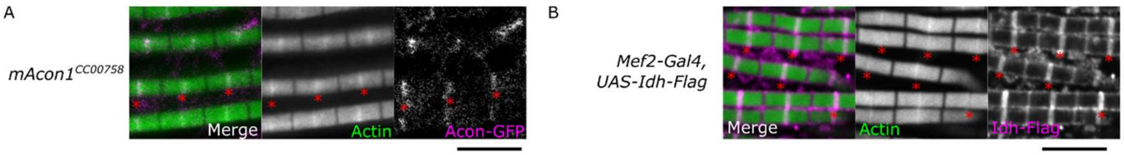

Fig. S6. Aconitase and Isocitrate dehydrogenase localize to the Z-disc. (A) Confocal image of the IFM carrying the Acon1-GFP protein trap (*mAcon1^{cc00758}).* A faint GFP signal is observed at the Z-disc (red asterisks). GFP is shown in magenta (B) Confocal image of the IFM overexpressing Idh-Flag (*Mef2-Gal4; UAS-Idh-Flag*) and stained with anti-flag. A strong Flag signal is observed at the Z-disc. Flag staining is shown in magenta. In both panels, actin filaments are shown in green. The scale bar is 5 µm.

Table S1. Summary of myofibril phenotypes observed by the depletion of TCA components using RNAi with Act88f-Gal4

Enzyme name	Drosophila short name	RNAi library	RNAi line ID	Phenotype
Aconitase	mAcon1	KK	KK103809	Severe
Isocitrate dehydrogenase	Idh3b	KK	KK102960	Normal
Isocitrate dehydrogenase	Idh3b	GD	GD6219	Strong/Severe
Isocitrate dehydrogenase	Idh3a/l(1)G0156	GD	P{GD5222}v41191	Normal
Isocitrate dehydrogenase	Idh3a/l(1)G0156	GD	P{GD5222}v41192	Weak/Mild
Isocitrate dehydrogenase	Idh3a/l(1)G0156	GD	P{GD16641}v50828	Weak
Isocitrate dehydrogenase	Idh3a/l(1)G0156	KK	KK107912	Strong/Severe
Isocitrate dehydrogenase	CG5028	GD	GD6271	Strong
Isocitrate dehydrogenase	CG5028	KK	KK102781	Strong
ATP citrate lyase	ATPCL	HM	HMC06049	Normal/weak
Fumarase 1	Fum1/CG4094	KK	KK108008	Normal
Fumarase 1	Fum1/CG4094	HM	HMC03334	Normal/Weak
Cytochrome c oxidase subunit 5A	Cox5	HM	HMJ22367	Normal
Cytochrome c oxidase subunit 5A	Cox5	JF	JF02700	Normal
Dihydrolipoamide succinyltransferase E2 subunit	DLST/CG5214	HM	HMC03051	Mild/Strong
Dihydrolipoamide succinyltransferase E2 subunit	DLST/CG5214	KK	KK109081	Weak
Dihydrolipoamide dehydrogenase E3 subunit	DLD/CG7430	KK	KK102614	Strong
Citrate synthase	kdn	KK	KK107737	Weak
Malate dehydrogenase 2	mdh2	KK	KK109040	Mild
Malate dehydrogenase 1	mdh1	KK	KK108844	Normal
Succinyl-coenzyme A synthetase β subunit, ADP-forming	ScsβA/skap	KK	KK101171	Normal
Succinyl-coenzyme A synthetase α subunit 1	Scsα1	KK	KK102542	Strong/Severe
Succinyl-coenzyme A synthetase β subunit, GDP-forming	ScsβG/SucB	KK	KK109063	Normal

Normal: wild type; **Weak**: unfrequent and mild defects; **Mild**: Common defects; **Strong**: Common sarcomere dissgregation; **Severe**: No sarcomere structure

Table S2. A detailed list of strains and reagents.

Reagent type (species) or resource	Designation	Source or reference	Identifiers	Additional information
gene (*Drosophila melanogaster*	Zasp52		FBgn0265991	
gene (*Drosophila melanogaster*	Zasp66		FBgn0035917	
gene (*Drosophila melanogaster*	Actinin		FBgn0000667	
gene (*Drosophila melanogaster*	Nc73EF/OGDH		FBgn0010352	E1 subunit
gene (*Drosophila melanogaster*	DLST/CG5214		FBgn0037891	E2 subunit
gene (*Drosophila melanogaster*	DLD/CG7430		FBgn0036762	E3 subunit
genetic reagent (*Drosophila melanogaster*	*Act88F-Gal4*	RM Cripps PMID: 22975331	FBal0268407	
genetic reagent (*Drosophila melanogaster*	*OGDH-TRiP.HMS00554*	BDSC	RRID: BDSC_33686	RNAi against OGDH
genetic reagent (*Drosophila melanogaster*	*OGDH[MI06026-GFSTF.1]*	BDSC	59416	OGDH-GFP
genetic reagent (*Drosophila melanogaster*	*OGDH-GD.50393*	VDRC	RRID:VDRC_50393	*OGDH-GD.50393*
genetic reagent (*Drosophila melanogaster*	*UAS-LacZ*	BDSC	RRID:BDSC_3356	
genetic reagent (*Drosophila melanogaster*	*Zasp52-MI02988-mCherry*	Nicanor Gonzalez Morales PMID: 29423427	PMID:29423427	Replacement of the MIMIC02988 cassette in Zasp52 with an in-frame mCherry tag.
genetic reagent (*Drosophila melanogaster*	*Zasp52-GFP Zasp52[ZCL423]*	BDSC	RRID:BDSC_58790	
genetic reagent (*Drosophila melanogaster*	*Zasp66-GFP Zasp66[ZCL0663]*	BDSC	RRID:BDSC_6824	
genetic reagent (*Drosophila melanogaster*	*Obscurin-GFP*			
genetic reagent (*Drosophila melanogaster*	*Sls-GFP*			
genetic reagent (*Drosophila melanogaster*	*Zasp52[MI02988]*	BDSC	RRID:BDSC_41034	
genetic reagent (*Drosophila melanogaster*	*Zasp52[MI00979]*	BDSC	RRID:BDSC_33099	
genetic reagent (*Drosophila melanogaster*	*UAS-OGDH-NYFP*	Current study	N/A	The OGDH-PA isoform fused to NYFP.
genetic reagent (*Drosophila melanogaster*	*UAS-OGDH-BM-NYFP*	Current study	N/A	OGDHΔ741-769 mutant fused to NYFP.
genetic reagent (*Drosophila melanogaster*	*UAS-OGDH-wt-Venus*	Current study	N/A	Wild type OGDH-PA fused to Venus

genetic reagent (*Drosophila melanogaster*	*UAS-OGDH-BM-Venus*	Current study	N/A	OGDHΔ741-769 mutant fused to Venus
genetic reagent (*Drosophila melanogaster*	*UAS-human-OGDH-Flag*	H.Bellen PMID: 28017472	FBal0325019	Human OGDH fused to Flag tag.
genetic reagent (*Drosophila melanogaster*	*UAS-Drosophila-OGDH-Flag*	H.Bellen PMID: 28017472	FBst0077505	Drosophila OGDH fused to Flag tag
genetic reagent (*Drosophila melanogaster*	*UAS-Zasp52-PK-NYFP (CYFP)*	Nicanor Gonzalez Morales PMID: 31746737	N/A	The Zasp52-PK isoform fused to either NYFP or CYFP.
genetic reagent (*Drosophila melanogaster*	*UAS-Zasp52-PK-NYFP (CYFP)*	Nicanor Gonzalez Morales PMID: 31746737	N/A	
genetic reagent (*Drosophila melanogaster*	*OGDH-wt-mCh*	Current study	N/A	Endogenous OGDH tagged with His and mCherry.
genetic reagent (*Drosophila melanogaster*	*OGDH-BM-mCh*	Current study	N/A	Endogenous OGDH with a Δ741-769 and tagged with His and mCherry
genetic reagent (*Drosophila melanogaster*	*OGDH-ED-mCh*	Current study	N/A	Endogenous OGDH with H306A and H344A replacement mutations and tagged with His and mCherry
genetic reagent (*Drosophila melanogaster*	OGDH[T2A]	BDSC	RRID:BDSC_77497	Insertion mutant that introduces an early stop and likely represents a null mutant.Known as OGDH[MI06026-TG4.1]
genetic reagent (*Drosophila melanogaster*	*UAS-Cas9*	BDSC	RRID:BDSC_54595	
genetic reagent (*Drosophila melanogaster*	*OGDH[TKO.GS00550]*	BDSC	RRID:BDSC_576381	Guide RNA targeting OGDH
genetic reagent (*Drosophila melanogaster*	*DLD[TKO.GS00548]*	BDSC	RRID:BDSC_576379	Guide RNA targeting DLD/CG7430
genetic reagent (*Drosophila melanogaster*	*DLST[TKO.GS03432]*	BDSC	RRID:BDSC_583750	Guide RNA targeting DLST/CG5214
genetic reagent (*Drosophila melanogaster*	*DLD-GFP*	VDRC	318906	fTRG fosmid containinggenomic DLD tagged with EGFP and FLAG
genetic reagent (*Drosophila melanogaster*	*DLST-GFP*	Current study	N/A	fTRG fosmid containinggenomic DLST tagged with EGFP and FLAG
genetic reagent (*Drosophila melanogaster*	*UAS-RA.CS2*	BDSC	RRID:BDSC_538624	Cold sensitive Ricin toxin
genetic reagent (*Drosophila melanogaster*	*UAS-RpS5a-Venus*	Paul Lasko PMID: 31551467	FBal0355313	Ribosome GFP
Strains with RNAi targeting different TCA cycle enzymes				

Journal of Cell Science • Supplementary information

genetic reagent (*Drosophila melanogaster*	*mAcon1-KK103809*	VDRC	RRID:VDRC_10 3809
genetic reagent (*Drosophila melanogaster*	*Idh3b -KK102960*	VDRC	RRID:VDRC_10 2960
genetic reagent (*Drosophila melanogaster*	*Idh3b-GD6219*	VDRC	RRID:VDRC_14 443
genetic reagent (*Drosophila melanogaster*	*Idh3a-P{GD5222}v41191*	VDRC	RRID:VDRC_*41 191*
genetic reagent (*Drosophila melanogaster*	*Idh3a/-P{GD5222}v41192*	VDRC	RRID:VDRC_*41 192*
genetic reagent (*Drosophila melanogaster*	*Idh3a-P{GD16641}v50828*	VDRC	RRID:VDRC_*50 828*
genetic reagent (*Drosophila melanogaster*	*Idh3a-KK107912*	VDRC	RRID:VDRC_*10 7912*
genetic reagent (*Drosophila melanogaster*	*CG5028-GD6271*	VDRC	RRID:VDRC_52 043
genetic reagent (*Drosophila melanogaster*	*CG5028-KK102781*	VDRC	RRID:VDRC_*10 2781*
genetic reagent (*Drosophila melanogaster*	*Fum1-KK108008*	VDRC	RRID:VDRC_*10 8008*
genetic reagent (*Drosophila melanogaster*	*Fum1-HMC03334*	BDSC	RRID:BDSC_51 779
genetic reagent (*Drosophila melanogaster*	*Cox5A-HMJ22367*	BDSC	RRID:BDSC_58 282
genetic reagent (*Drosophila melanogaster*	*Cox5A-JF02700*	BDSC	RRID:BDSC_27 548
genetic reagent (*Drosophila melanogaster*	*DLST-HMC03051*	BDSC	RRID:BDSC_50 650
genetic reagent (*Drosophila melanogaster*	*DLST-KK109081*	VDRC	RRID:VDRC_*10 9081*
genetic reagent (*Drosophila melanogaster*	*DLD-KK102614*	VDRC	RRID:VDRC_*10 2614*
genetic reagent (*Drosophila melanogaster*	*kdn-KK107737*	VDRC	RRID:VDRC_*10 7737*
genetic reagent (*Drosophila melanogaster*	*mdh2-KK109040*	VDRC	RRID:VDRC_*10 9040*
genetic reagent (*Drosophila melanogaster*	*mdh1-KK108844*	VDRC	RRID:VDRC_*10 8844*
genetic reagent (*Drosophila melanogaster*	*skap-KK101171*	VDRC	RRID:VDRC_*10 1171*
genetic reagent (*Drosophila melanogaster*	*Scsα1-KK102542*	VDRC	RRID:VDRC_*10 2542*
genetic reagent (*Drosophila melanogaster*	*ScsβG-KK109063*	VDRC	RRID:VDRC_*10 9063*
recombinant DNA reagent	Plasmid: pGADT7	Clontech	630442
recombinant DNA reagent	Plasmid: pENTRY-Zasp52-PK	DGRC	DGRC: GEO02280
recombinant DNA reagent	Plasmid: pENTRY-Zasp52-PE GEO12859	DGRC	DGRC: GEO12859

recombinant DNA reagent	Plasmid: pENTRY-OGDH-PA GEO09867	DGRC	DGRC: GEO09867	
recombinant DNA reagent	FlyFos031562(pRedFlp-Hgr)(CG5214[18165]::S000169_fly_pretag)::2XTY1-SGFP-V5-preTEV-BLRP-3XFLAGdFRT	TransgeneOme	9680756374385061 A03	Flyfos clone containing DLST tagged with EGFP
recombinant DNA reagent	Plasmid: pGBKT7-GW	Addgene	61703	
recombinant DNA reagent	Plasmid: pGADT7-GW	Addgene	61702	
recombinant DNA reagent	Plasmid: pGBKT7- Zasp52 individual domains: PDZ, ZM, LIM1a, LIM1b, LIM2a, LIM2b, LIM3 and LIM4	Nicanor Gonzalez Morales PMID: 31746737	N/A	All individual domains of Zasp52 cloned into pGBKT7.
recombinant DNA reagent	Plasmid: pGBKT7GW-Zasp52-PK	Nicanor Gonzalez Morales PMID: 31746737	N/A	Zasp52-PK isoform cloned into pGBKT7GW using Gateway cloning.
recombinant DNA reagent	Plasmid: pGADT7GW-Zasp52-PK	Nicanor Gonzalez Morales PMID: 31746737	N/A	Zasp52-PK isoform cloned into pGADT7GW using Gateway cloning.
recombinant DNA reagent	Plasmid: pGBKT7GW-Zasp52-PE	Nicanor Gonzalez Morales PMID: 31746737	N/A	Zasp52-PE isoform cloned into pGBKT7GW using Gateway cloning
recombinant DNA reagent	Plasmid: pGADT7GW-Zasp52-PE	Nicanor Gonzalez Morales PMID: 31746737	N/A	Zasp52-PE isoform cloned into pGADT7GW using Gateway cloning.
recombinant DNA reagent	Plasmid: pDEST- pUAS-RfB-HA-CYFP-attB	Sven Bogdan PMID: 20937809	FBrf0212496	
recombinant DNA reagent	Plasmid: pDEST- pUAS-RfB-myc-NYFP-attB	Sven Bogdan PMID: 20937809	FBrf0212496	
strain, strain background (*Saccharomyces cerevisiae*)	Matchmaker Y2HGold	Clontech	630498	
strain, strain background (*E. coli*)	BL21	NEB	C2530H	
software, algorithm	R Project for Statistical Computing: base and ape packages	cran.r-project.org	RRID:SCR_001905	
software, algorithm	ImageJ/Fiji distribution	https://fiji.sc/	RRID: SCR_002285	
chemical compound, drug	YPDA medium	Clontech	630464	
chemical compound, drug	Minimal SD Base	Clontech	630411	
chemical compound, drug	-Leu/-Trp DO Supplement	Clontech	630417	

Journal of Cell Science • Supplementary information

chemical compound, drug	-Ade/-His/-Leu/-Trp DO Supplement	Clontech	630428
chemical compound, drug	Acti-stain 488 phalloidin	CYTOSKELETON, INC	PHDG1-A
chemical compound, drug	Alexa633-Phalloidin	Fisher Scientific	A22284
chemical compound, drug	Rhodamine-phalloidin	Fisher Scientific	10063052
chemical compound, drug	Monoclonal anti-flag	Sigma	F3165
