# Supplementary material for: The oxoglutarate dehydrogenase complex is involved in myofibril growth and Z-disc assembly in Drosophila
Source: J Cell Sci. 2023 Jun 30;136(13):jcs260717. doi: 10.1242/jcs.260717 (PMC10323237; doi:10.1242/jcs.260717)
Supplement: Supplementary file 1 [file joces-136-260717_review_history.pdf]

The oxoglutarate dehydrogenase complex is involved in myofibril and Z-disc assembly in *Drosophila*

Nicanor González Morales, Oceane Marescal, Szilárd Szikora, Anja Katzemich, Tuana Correia-Mesquita, Peter Bıró, Miklos Erdelyi, József Mihaly and Frieder Schöck
DOI: 10.1242/jcs.260717

Editor: John Heath

Review timeline
Original submission: 17 October 2022
Editorial decision: 30 December 2022
First revision received: 2 May 2023
Accepted: 24 May 2023

Original submission

First decision letter

MS ID#: JOCES/2022/260717

MS TITLE: Oxoglutarate dehydrogenase coordinates myofibril growth by maintaining amino acid homeostasis

AUTHORS: Nicanor Gonzalez-Morales, Oceane Marescal, Szilard Szikora, Anja Katzemich, Peter Biro, Miklos Erdelyi, Tuana Mesquita, Jozsef Mihaly, and Frieder Schoeck
ARTICLE TYPE: Research Article

We have now reached a decision on the above manuscript.

To see the reviewers' reports and a copy of this decision letter, please go to: https://submit-jcs.biologists.org and click on the 'Manuscripts with Decisions' queue in the Author Area. (Corresponding author only has access to reviews.)

As you will see, the reviewers raise a number of substantial criticisms that prevent me from accepting the paper at this stage. They suggest, however, that a revised version might prove acceptable, if you can address their concerns. In particular you will need to include extra data to convince the referees of the specificity of your findings. If you think that you can deal satisfactorily with the criticisms on revision, I would be pleased to see a revised manuscript. We would then return it to the reviewers.

Please ensure that you clearly highlight all changes made in the revised manuscript. Please avoid using 'Tracked changes' in Word files as these are lost in PDF conversion.

I should be grateful if you would also provide a point-by-point response detailing how you have dealt with the points raised by the reviewers in the 'Response to Reviewers' box. Please attend to all of the reviewers' comments. If you do not agree with any of their criticisms or suggestions please explain clearly why this is so.

© 2023. Published by The Company of Biologists under the terms of the Creative Commons Attribution License (https://creativecommons.org/licenses/by/4.0/).

Reviewer 1

Advance summary and potential significance to field

In the manuscript by Gonzalez Morales et al, the authors investigate the role of oxoglutarate dehydrogenase (OGDH), an enzyme in the TCA cycle normally found in the mitochondria, here in myofibril growth and development in the fly indirect flight muscle (IFM). The authors find that OGDH binds to LIM domains of ZASP and in doing so localizes to Z-discs of the IFM. Overall, the data show that OGDH does indeed localize to z-discs, and that loss of function of OGDH results in abrogation of proper myofibril development and/or altered z-disc morphology.

However, there are a few larger concerns about the specificity of this activity for myofibril development per se, and the requirement for Z-disc localization of the TCA machinery for myofibril development. The concerns are listed below in major and minor categories.

Comments for the author

Major concerns:
1) One larger concern is that while it is clear that TCA cycle activity is important for muscle development, it is likely essential for most tissue development in embryogenesis, so it leads to the question of how specific and proximal this effect is, especially in their null/crispr mutants. Is it not the case that most of the embryos tissues show developmental defects? If so, then how does this shed light on specific mechanism of myofibrillogenesis other than it needs amino acids to occur? Is there a way to limit gRNA expression to only developing muscle? Even so, this would be a fairly small intellectual step forward, given that all cells will require amino acids and protein synthesis to occur for normal development.

2) The second larger concern is that while OGDH clearly localizes to Z-discs and binds ZASP, the data do not show that this localization is important for myofibril development. The ZASP binding defective mutant appears to have completely normal z-discs and myofibril organization. In addition, it seems as though the z-disc localized OGDH is a tiny fraction of the total pool of this enzyme, as the authors point out the much more intense signal from the mitochondria (as one might expect, shown in Figure S3). Given the broad function of the TCA machinery, this would suggest that the z-disc localization may not be immediately relevant for OGDH function in myofibrillogenesis. Are the OGDH-BM flies impaired in flight or subsequent muscle development?

Minor Comments:

3) The rationale for picking out OGDH from their ERC analysis was not very clear- several proteins show up in this analysis with a higher ERC value to ZASP. If this selection was based on a myofibril localization screen, that might be helpful to show to make that point.

4) There are a few typos in the figure legends and text, a re-review of the text would be helpful.

Reviewer 2

Advance summary and potential significance to field

The manuscript from Gonzalez-Morales et al describes an interesting study, which strives to decipher an intriguing and unexpected connection the authors establish between the TCA cycle element OGDH and growth of body-wall muscle sarcomeres of Drosophila larvae.

In their study, the authors use a wide range of experimental approaches, which they employ in competent fashion. They initially identify the OGDH-E1 subunit of the OGDH protein complex as a potential interacting partner of Zasp family proteins, key sarcomeric elements which the Schock lab has studied extensively.

© 2023. Published by The Company of Biologists under the terms of the Creative Commons Attribution License (https://creativecommons.org/licenses/by/4.0/).

The authors proceed to demonstrate that- similar to Zasps- OGDH localizes to sarcomere Z-discs (in addition to its known mitochondrial localization) and is required for myofibril growth. Additional data is then presented and used to make several substantial claims regarding OGDH function: that OGDH is recruited to Z-discs by Zasp; that the enzymatic activity of OGDH is required for its muscle function; and that the OGDH complex- whose spatial organization at the Z-disc is visualized using super-resolution microscopy- contributes to myofibril growth by maintaining an amino acid balance that is necessary for proper levels of protein synthesis.

Comments for the author

While the initial set of experiments provides a solid basis for the study (but see some points below), various aspects of the analysis designed to probe and comprehend the functional link between a metabolic enzyme and myofiber morphology and growth are, to my mind, less compelling, and in need of further action and elaboration as detailed below:

1. The ages of the flies from which IFM samples were prepared are generally missing- they should be mentioned in the figure legends.

2. In the absence of anti-OGDH antibody staining, localization of OGDH-GFP (Figure 1B) is the key observation behind the claim of Z-disc localization of OGDH. The authors justifiably seek to back up this claim with additional data (Fig S2). More convincing to my mind are the observations that two additional GFP-tagged subunits of the OGDH complex localize to the Z-disc (Figure 4 H-L), and I would strongly recommend that these observations be moved up to Figure 1.

3. OGDH-mCherry construct experiments:

- It is stated (line 202) that the OGDH-mCherry constructs “strongly localize to the mitochondria (Fig S3)”.
How is this determined? Strong fluorescence is indeed observed between myofibrils, but what is the basis for asserting that it localizes to mitochondria ?

- The OGDH-BM-mCherry variant is stated to be homozygous lethal (line 204). This is a potentially informative finding. What is the nature of this lethality? Are muscles produced by these flies and if so, do they show a myofibril growth phenotype?

- Localization of the OGDH-BM-mCherry variant to Z-discs, although diminished relative to intact OGDH-mCherry, is somewhat surprising given the data reported earlier in the manuscript. The authors should test and report how these variants localize in the two Zasp52 mutant backgrounds used to study OGDH-GFP localization.

As noted above, the more problematic portion of the manuscript relates to the (important) effort of explaining how a TCA cycle element could contribute to myofibril growth:

4. The question of whether the enzymatic activity of OGDH is required for its muscle-related functions is clearly an important one in this context. The authors assert that their findings support the notion that OGDH enzymatic activity is required, but I find the data presented not to be conclusive. The observation of similar disruptions to myogenesis following knockdown of all three subunits of the OGDH complex (Figure 4D-G) is indeed consistent with a requirement for enzymatic activity. However, a much more extensive analysis than what is provided (Figure 4A-C) is necessary in order to persuasively claim that both full knockout and the “enzyme dead” variant of OGDH are similarly required for embryonic myogenesis, and specifically for myofibril growth. The entire issue of enzymatic activity rests, to my mind, on rather shaky ground and that- at best- should be brought up as a possibility that requires further study.

5. The authors demonstrate similar myofibril knockdown phenotypes for most TCA elements that they examine, consistent with a requirement for the entire cycle during IFM myogenesis. What seems to be missing is data regarding localization of these elements. This is significant in the context of addressing whether OGDH (and, by inference, the entire cycle) function within

© 2023. Published by The Company of Biologists under the terms of the Creative Commons Attribution License (https://creativecommons.org/licenses/by/4.0/).

sarcomeres or- more indirectly- at mitochondria, an issue that the authors properly bring up at the beginning of the Discussion section.

6. The authors provide evidence that generation of ATP by the TCA cycle seems not to be required for its role in myogenesis, and consequentially put forward a metabolomic-based analysis which leads them to suggest that the TCA cycle regulates the balance of amino acids, which in turn affects protein synthesis and, ultimately, myofibril growth. This is an attractive idea, and the authors do provide data consistent with this notion, but it remains highly speculative. Care should therefore be taken with its presentation- it certainly cannot be used to make such definitive statements as those that appear in the title and the concluding sentence of the abstract.

Minor points:

The description of striated muscle, myofibril and sarcomere structure and function in the first half of the Introduction section relies almost exclusively on references related to Drosophila myogenesis. The authors should make an effort to include non-Drosophila studies and reviews among the publications cited, given that much of what is described holds true for vertebrate muscles, and that the current study focuses on conserved elements and therefore may well be of general relevance.

7. Figure 1 legend:
(B)- please clarify here that the OGDH[MI06026-GFSTF.1] fly line is a GFP “protein trap”- otherwise it is not clear where “OGDH-GFP” comes from.
(D)- what is the arrow pointing to in the panel?

8. Supplementary figures S1 and S2 should be re-labeled, so that they appear in order in the text.

9. For clarification purposes, the sentence (line 178) “We have previously shown that the LIM domains of
Zasp52 bind to the ZM domains” should be extended and state “of Zasp proteins”.

10. Figure 4 (H-L) legend. As mentioned above, it is recommended that this data appear as part of Figure 1. In addition:
- panels K,L are not described in the legend. Furthermore, the single-channel panels should be shown in addition to the merged channel view.

- it is not readily apparent to readers that the different OGDH subunit elements are visualized via GFP tags. This is mentioned as part of the STORM microscopy section, but should be stated clearly in the figure legend and perhaps also in the text. Furthermore- where does the DLD-GFP line come from?

First revision

<u>Author response to reviewers' comments</u>

We thank the reviewers for their insights, which have greatly helped us to better understand the phenotype. We provide a better and more careful interpretation of the phenotypes we observe that is more in line with the observed phenotypes. We added important new data, in particular additional evidence for a Z-disc specific defect in OGDH mutants (Fig. 2) and removed the embryonic data because it did not clarify the function of OGDH. Below we provide a point-by-point response.

Reviewer 1 Advance Summary and Potential Significance to Field:
In the manuscript by González Morales et al, the authors investigate the role of oxoglutarate dehydrogenase (OGDH), an enzyme in the TCA cycle normally found in the mitochondria, here in myofibril growth and development in the fly indirect flight muscle (IFM). The authors find that

© 2023. Published by The Company of Biologists under the terms of the Creative Commons Attribution License (https://creativecommons.org/licenses/by/4.0/).

OGDH binds to LIM domains of ZASP and in doing so localizes to Z-discs of the IFM. Overall, the data show that OGDH does indeed localize to z-discs, and that loss of function of OGDH results in abrogation of proper myofibril development and/or altered z-disc morphology. However, there are a few larger concerns about the specificity of this activity for myofibril development per se, and the requirement for Z-disc localization of the TCA machinery for myofibril development. The concerns are listed below in major and minor categories.

Reviewer 1 Comments for the Author:

Major concerns:

1)One larger concern is that while it is clear that TCA cycle activity is important for muscle development, it is likely essential for most tissue development in embryogenesis, so it leads to the question of how specific and proximal this effect is, especially in their null/crispr mutants. Is it not the case that most of the embryos tissues show developmental defects? If so, then how does this shed light on specific mechanism of myofibrillogenesis other than it needs amino acids to occur? Is there a way to limit gRNA expression to only developing muscle? Even so, this would be a fairly small intellectual step forward, given that all cells will require amino acids and protein synthesis to occur for normal development.

We agree that the embryo data does not show the specificity of OGDH function in developing muscles. For the embryo experiments, we used OGDH alleles ($OGDH^{t2a}$, $OGDH^{Binding\ mutant}$, and $OGDH^{Enzyme\ dead}$), that are all homozygous lethal at the embryo stage. Therefore we cannot test them in adult muscles, and we decided to remove all embryonic data from the current paper.

Nevertheless, in Figure 5 a-d we show the muscle defects that arise from depleting the OGDH complex subunits specifically in developing Indirect flight muscles by expressing Cas9 protein in the IFM and gRNA for either OGDH, DLD, or DLST. The RNAi phenotypes are also tissue specific. In these cases, other tissues are not affected.

We propose a dual role of OGDH: a global provision of amino acids likely mediated by mitochondrial metabolism, which leads to some generally smaller myofibrils often observed in some TCA mutants. And a local provision of amino acids owing to the Z-disc localization of OGDH, which results in a specific Z-disc growth or assembly defect, but not in M-line defects (new Fig. 2). In addition, we show that OGDH is linked to amino acid homeostasis rather than ATP synthesis during myofibril growth (perturbing oxidative phosphorylation has no effect on myofibril development (Fig. 6B).

The main point is that if OGDH had exclusively a mitochondrial function (providing global amino acids), we should only see smaller myofibrils, but not small Z-discs within relatively normal-sized myofibrils. Disruption of global protein synthesis by ricin expression (Fig. 8) results in smaller myofibrils, but with normal Z-discs and M-line (myofibril growth is disrupted, but not myofibril assembly and Z-disc structure).

2) The second larger concern is that while OGDH clearly localizes to Z-discs and binds ZASP, the data do not show that this localization is important for myofibril development. The ZASP binding defective mutant appears to have completely normal z-discs and myofibril organization. In addition, it seems as though the z-disc localized OGDH is a tiny fraction of the total pool of this enzyme, as the authors point out the much more intense signal from the mitochondria (as one might expect, shown in Figure S3). Given the broad function of the TCA machinery, this would suggest that the z-disc localization may not be immediately relevant for OGDH function in myofibrillogenesis. Are the OGDH-BM flies impaired in flight or subsequent muscle development?

One of the major points of our work is that OGDH localizes to the Z-disc, which is unexpected. We show enough evidence to support the claim.

The Zasp binding defective mutant ($OGDH^{BM}$) is homozygous lethal at embryonic stages. We therefore cannot separate the mitochondrial function from the Z-disc function in IFM with this mutant. A major analysis of the requirements of OGDH in embryonic muscles would take a significant amount of time and would not answer the issue of the function of OGDH

© 2023. Published by The Company of Biologists under the terms of the Creative Commons Attribution License (https://creativecommons.org/licenses/by/4.0/).

at the Z-disc in the flight muscles. We have therefore removed all embryonic data from the paper.

It is true that in the IFM most of the OGDH molecules are in the mitochondria and only a fraction is in the Z-disc. As mentioned above, we show a specific effect only on the Z-disc, not the M-line (Fig. 2D). This strongly suggests that the function of OGDH is not just the general production of amino acids for the entire flight muscle, but that it is required at the Z-disc. Disruption of all protein synthesis by ricin expression (Fig. 8, which is very similar to removing the entire amino acid pool) results in smaller myofibrils, but with normal Z-discs and M-line. In addition, we show that the other subunits of the complex interact with OGDH at the Z-disc (dSTORM data), and that the two enzymes that directly precede OGDH in the TCA cycle also localize to the Z-disc (new supplementary figure 6).

We do not question the mitochondrial role of the TCA cycle or the OGDH complex. We are just adding that some of these enzymes are components of the Z-disc, which suggest a function outside the mitochondria. We agree that the mitochondrial pool of OGDH likely also contributes to a certain extent to the sarcomere phenotype (resulting in somewhat smaller myofibrils in some of the TCA mutants).

The OGDH complex has also been found in the nucleus, where it has a role in chromatin regulation. In those cells the majority of the OGDH molecules are in the mitochondria and only a tiny fraction in the nucleus. That does not mean that the nuclear fraction is not functional.

We more clearly communicate throughout the text the limitations of our analysis with respect to mitochondrial and the Z-disc function.

Minor Comments:
3)The rationale for picking out OGDH from their ERC analysis was not very clear- several proteins show up in this analysis with a higher ERC value to ZASP. If this selection was based on a myofibril localization screen, that might be helpful to show to make that point. 4)There are a few typos in the figure legends and text, a re-review of the text would be helpful.

OGDH was the candidate with the most obvious Z-disc localization pattern of the ones we screened for.

Reviewer 2 Advance Summary and Potential Significance to Field:
The manuscript from Gonzalez-Morales et al describes an interesting study, which strives to decipher an intriguing and unexpected connection the authors establish between the TCA cycle element OGDH and growth of body-wall muscle sarcomeres of Drosophila larvae.

In their study, the authors use a wide range of experimental approaches, which they employ in competent fashion. They initially identify the OGDH-E1 subunit of the OGDH protein complex as a potential interacting partner of Zasp family proteins, key sarcomeric elements which the Schock lab has studied extensively. The authors proceed to demonstrate that- similar to Zasps- OGDH localizes to sarcomere Z-discs (in addition to its known mitochondrial localization) and is required for myofibril growth. Additional data is then presented and used to make several substantial claims regarding OGDH function: that OGDH is recruited to Z-discs by Zasp; that the enzymatic activity of OGDH is required for its muscle function; and that the OGDH complex- whose spatial organization at the Z-disc is visualized using super-resolution microscopy- contributes to myofibril growth by maintaining an amino acid balance that is necessary for proper levels of protein synthesis.

Reviewer 2 Comments for the Author:
While the initial set of experiments provides a solid basis for the study (but see some points below), various aspects of the analysis designed to probe and comprehend the functional link between a metabolic enzyme and myofiber morphology and growth are, to my mind, less compelling, and in need of further action and elaboration as detailed below:

1. The ages of the flies from which IFM samples were prepared are generally missing- they should be mentioned in the figure legends.

© 2023. Published by The Company of Biologists under the terms of the Creative Commons Attribution License (https://creativecommons.org/licenses/by/4.0/).

We used flies that are 1 or 2 days old, to avoid muscle degeneration defects. We have added a detailed sentence in the materials section, and we have added a brief statement in all the figure legends.

2. In the absence of anti-OGDH antibody staining, localization of OGDH-GFP (Figure 1B) is the key observation behind the claim of Z-disc localization of OGDH. The authors justifiably seek to back up this claim with additional data (Fig S2). More convincing to my mind are the observations that two additional GFP-tagged subunits of the OGDH complex localize to the Z-disc (Figure 4 H-L), and I would strongly recommend that these observations be moved up to Figure 1.
We moved OGDH-mCHerry to figure 1

Thank you for the suggestion. We have made new figures 1 and 2. The new Fig. 1 contains the Z-disc localization data from conformal microscopy OGDH-GFP and from dSTROM super resolution microscopy of OGDH, DLD, and DLST. We agree that having the dSTORM data in Figure 1 strengthens the point that the OGDH complex is a Z-disc component. We have added that GFP is used as a tag for all the subunits in the text and in the figure legend. It is also in the materials and methods section. Figure 2 now contains the phenotypic characterization of OGDH-RNAi.

3. OGDH-mCherry construct experiments:
- It is stated (line 202) that the OGDH-mCherry constructs “strongly localize to the mitochondria (Fig S3)”.
How is this determined? Strong fluorescence is indeed observed between myofibrils, but what is the basis for asserting that it localizes to mitochondria ?

The IFM have a high mitochondrial density and they are arranged in the intermyofibrillar space. The mitochondrial pattern in the IFM is very distinctive , other organelles have different patterns. See for example, Katti P, et al. Exp Cell Res. 2021 PMID: 33450208 and Avellaneda J, et al. Nat Commun. 2021 Apr 7;12(1):2091. PMID: 33828099; PMCID: PMC8027795.

- The OGDH-BM-mCherry variant is stated to be homozygous lethal (line 204). This is a potentially informative finding. What is the nature of this lethality? Are muscles produced by these flies and if so, do they show a myofibril growth phenotype?

We have removed embryonic data from the paper (see comments for reviewer 1).

- Localization of the OGDH-BM-mCherry variant to Z-discs, although diminished relative to intact OGDH-mCherry, is some iwhat surprising given the data reported earlier in the manuscript. The authors should test and report how these variants localize in the two Zasp52 mutant backgrounds used to study OGDH-GFP localization.

We did not do this experiment, because the Z-disc signal of OGDH-BM is already very weak in wild type. Some remaining Z-disc signal in OGDH-BM is not surprising: it could reflect an incomplete disruption of binding between OGDH-BM and Zasp52 or the recruitment of OGDH-BM to the Z-disc by the other proteins of the OGDH complex.

4. The question of whether the enzymatic activity of OGDH is required for its muscle-related functions is clearly an important one in this context. The authors assert that their findings support the notion that OGDH enzymatic activity is required, but I find the data presented not to be conclusive. The observation of similar disruptions to myogenesis following the knockdown of all three subunits of the OGDH complex (Figure 4D-G) is indeed consistent with a requirement for enzymatic activity. However, a much more extensive analysis than what is provided (Figure 4A-C) is necessary to persuasively claim that both full knockout and the “enzyme dead” variant of OGDH are similarly required for embryonic myogenesis, and specifically for myofibril growth. The entire issue of enzymatic activity rests, to my mind, on rather shaky ground and that- at best- should be brought up as a possibility that requires further study.

© 2023. Published by The Company of Biologists under the terms of the Creative Commons Attribution License (https://creativecommons.org/licenses/by/4.0/).

We agree that our interpretation of the OGDH-enzyme dead mutant was preliminary. We have removed the data corresponding to the mutant to avoid overinterpreting the results. Because the enzyme-dead mutants are lethal as homozygotes, we can not analyze the myofibril structure of the IFM and instead analyzed the larval body wall muscle. This approach assumes that both muscles have similar metabolic developments and growth patterns, which is unlikely given their developmental times and origins. Our follow-up experiments with the mutants using embryos have proven inconclusive, which we attribute to the differences between muscle types. In any case, we have removed the mutant data because it is inconclusive and does not add any information to the manuscript.

We still propose that the underlying defects we observed in IFM are metabolically based. The data that supports our claim are 1) the RNAi against other enzymes of the TCA cycle have similar phenotypes and 2) the CRISPR-Cas9 mutants of all the three subunits of the OGDH complex have very similar myofibril phenotypes. In addition, we have tested the Z-disc localization of other TCA cycle enzymes using available tools, and we found that two of them also localize to the Z-disc.

5. The authors demonstrate similar myofibril knockdown phenotypes for most TCA elements that they examine, consistent with a requirement for the entire cycle during IFM myogenesis. What seems to be missing is data regarding localization of these elements. This is significant in the context of addressing whether OGDH (and, by inference, the entire cycle) function within sarcomeres or- more indirectly- at mitochondria, an issue that the authors properly bring up at the beginning of the Discussion section.

We don't have the tools to test the localization of all the TCA cycle enzymes in the IFM. We have a GFP trap allele of Aconitase and a Flag-tagged version of Isocitrate dehydrogenase. We have analyzed and added their localization data to the manuscript (Supplementary Figure 6. They both localize to the Z-disc. In total, we present Z-disc localization data for 5 enzymes of the TCA cycle. The three subunits of the OGDH complex, Aconitase, and Isocitrate Dehydrogenase. The localization data does not rule out that the mitochondrial pool of OGDH is required for myofibril growth (as mentioned before we believe that some of the phenotypes - the smaller myofibrils, are due to reduced mitochondrial OGDH function), but it does tell that enzyme localization at the Z-disc is a common phenomenon and likely contributes to the Z-disc-specific defects we see.

6. The authors provide evidence that generation of ATP by the TCA cycle seems not to be required for its role in myogenesis, and consequentially put forward a metabolomic-based analysis which leads them to suggest that the TCA cycle regulates the balance of amino acids, which in turn affects protein synthesis. and, ultimately, myofibril growth. This is an attractive idea, and the authors do provide data consistent ith this notion, but it remains highly speculative. Care should therefore be taken with its presentation- it certainly, cannot be used to make such definitive statements as those that appear in the title and the concluding sentence of the abstract.

We apologize for overly enthusiastic statements; we have altered the text to be much more careful in our interpretations. We changed the abstract and discussion to account for a better interpretation.

Minor points:

The description of striated muscle, myofibril and sarcomere structure and function in the first half of the Introduction section relies almost exclusively on references related to Drosophila myogenesis. The uthors should make an effort to include non-Drosophila studies and reviews among the publications cited, given that much of what is described holds true for vertebrate muscles, and that the current study focuses on conserved elements and therefore may well be of general relevance.

We have added literature reviews focused on vertebrate sarcomeres.

1. *Dos Remedios, C. and D. Gilmour, An historical perspective of the discovery of titin filaments. Biophys Rev, 2017. 9(3): p. 179-188.*

© 2023. Published by The Company of Biologists under the terms of the Creative Commons Attribution License (https://creativecommons.org/licenses/by/4.0/).

2. *Ahmed, R.E., et al., Sarcomere maturation: function acquisition, molecular mechanism, and interplay with other organelles. Philos Trans R Soc Lond B Biol Sci, 2022. 377(1864): p. 20210325.*

7. Figure 1 legend:
(B)- please clarify here that the OGDH[MI06026-GFSTF.1] fly line is a GFP “protein trap”- otherwise it is not clear where “OGDH-GFP” comes from.

We corrected the legend.

(D)- what is the arrow pointing to in the panel?

A small protein aggregate, we added the description in the figure legend.

8. Supplementary figures S1 and S2 should be re-labeled, so that they appear in order in the text.

Yes, and we apologize for the mistake. We have corrected the numbering.

9. For clarification purposes, the sentence (line 178) “We have previously shown that the LIM domains of Zasp52 bind to the ZM domains” should be extended and state “of Zasp proteins”.

We corrected the statement.

10. Figure 4 (H-L) legend. As mentioned above, it is recommended that this data appear as part of Figure 1. In addition:
- panels K,L are not described in the legend. Furthermore, the single-channel panels should be shown in addition to the merged channel view.
- it is not readily apparent to readers that the different OGDH subunit elements are visualized via GFP tags. This is mentioned as part of the STORM microscopy section, but should be stated clearly in the figure legend and perhaps also in the text. Furthermore- where does the DLD-GFP line come from?

We show the single channel panels in the new figure 1 c-d

Both DLST-GFP and DLD-GFP lines are fosmid based. The fosmids carrying the GFP-tagged versions were originally described in Sarov, 2016 (doi: 10.7554/eLife.12068). For DLST, we obtained the fosmid and made a transgenic fly line with the fosmid inserted into attp40. The Dld line was already available at the VDRC stock center VDRC# 318906 (FlyFos029558(pRedFlp-Hgr)(CG7430[28617]::2XTY1-SGFP-V5-preTEV-BLRP-3XFLAG)dFRT). Originally we added information about the Drosophila strains we used in a Resource table. We have changed that to the material and method section to facilitate the interpretation of the results.

<u>Second decision letter</u>

MS ID#: JOCES/2022/260717

MS TITLE: The oxoglutarate dehydrogenase complex is involved in myofibril and Z-disc assembly in Drosophila

AUTHORS: Nicanor Gonzalez-Morales, Oceane Marescal, Szilard Szikora, Anja Katzemich, Peter Biro, Miklos Erdelyi, Tuana Mesquita, Jozsef Mihaly, and Frieder Schoeck
ARTICLE TYPE: Research Article

I am happy to tell you that your manuscript has been accepted for publication in Journal of Cell Science, pending standard ethics checks.

© 2023. Published by The Company of Biologists under the terms of the Creative Commons Attribution License (https://creativecommons.org/licenses/by/4.0/).

Reviewer 1

Advance summary and potential significance to field

In this revised manuscript, the authors show that oxoglutarate dehydrogenase (OGDH) is localized to the Z-lines of muscle, via its ability to bind directly to ZASP, another z-line protein. The authors go on to show that OGDH contributes to normal Z-line morphology and muscle function in specific muscle fibers (eg indirect flight muscle in flies).

Comments for the author

The revised manuscript has addressed all of my major concerns, and contains helpful new data to support their conclusions. Likewise, removal of some of the less easy to cleanly interpret data (embryonic data) has also strengthened the paper.

Reviewer 2

Advance summary and potential significance to field

The manuscript from Gonzalez-Morales et al describes an interesting study, which strives to decipher an intriguing and unexpected connection the authors establish between the TCA cycle element OGDH and growth of body-wall muscle sarcomeres of Drosophila larvae.

In their study, the authors use a wide range of experimental approaches, which they employ in competent fashion. They initially identify the OGDH-E1 subunit of the OGDH protein complex as a potential interacting partner of Zasp family proteins, key sarcomeric elements which the Schock lab has studied extensively. The authors proceed to demonstrate that- similar to Zasps- OGDH localizes to sarcomere Z-discs (in addition to its known mitochondrial localization) and is required for myofibril growth. Additional data is then presented and used to make several substantial claims regarding OGDH function: that OGDH is recruited to Z-discs by Zasp; that the enzymatic activity of OGDH is required for its muscle function; and that the OGDH complex- whose spatial organization at the Z-disc is visualized using super-resolution microscopy- contributes to myofibril growth by maintaining an amino acid balance that is necessary for proper levels of protein synthesis.

Comments for the author

In their revised manuscript and response to reviewer comments, the authors of the Gonzalez-Morales et al manuscript have made a good effort to address the critical comments and suggestions made in my review of their original submission. Some new, clarifying data and text have been added, but the main improvement comes from a considerably more focused and streamlined presentation of the findings and observations, and a toning down of the language used when providing interpretations.

© 2023. Published by The Company of Biologists under the terms of the Creative Commons Attribution License (https://creativecommons.org/licenses/by/4.0/).
